# Extracting homogenous data from heterogenous diseases: RaraSwed, the Swedish national rare disease quality registry

**DOI:** 10.1186/s44263-026-00276-9

**Published:** 2026-06-16

**Authors:** Sanna Mansoob, Dan Hellström, Magnus Burstedt, Malin Grände, Cecilia Gunnarsson, Stephanie Juran, Lovisa Lovmar, Katarina Sandgren, Maria Johansson Soller, Anna Wedell, Cecilia Soussi Zander, Alexandra Wennberg, Marie Stenmark Askmalm

**Affiliations:** 1 Department of Paediatrics, Skåne University Hospital, Southern Healthcare Region, Lund, Sweden; 2 Division of Paediatrics, Department of Clinical Sciences Lund, Lund University, Lund, Sweden; 3 Centre for Rare Disease South, Department of Genetics, Pathology and Molecular Diagnostics, Office for Medical Services, Southern Healthcare Region, Lund, Sweden; 4 National Committee for Knowledge-Driven Management for Rare Diseases (Nationella Programområdet Sällsynta Diagnoser) within the National System for Knowledge-Driven Management, Swedish Association of Local Authorities and Regions (SALAR), Stockholm, Sweden; 5 Department of Medical and Clinical Genetics, Institute of Medical Biosciences, Umeå University Hospital, Northern Healthcare Region, Umeå, Sweden; 6 Rare Diseases Sweden, National Patient Advocacy Group, Riksförbundet Sällsynta diagnoser, Sundbyberg, Sweden; 7 Department of Clinical Genetics, Linköping University Hospital, Southeast Healthcare Region, Linköping, Sweden; 8 Department of Clinical Genetics and Genomics, Sahlgrenska University Hospital, Western Healthcare Region, Göteborg, Sweden; 9 Department of Molecular Medicine and Surgery, Karolinska Institute, Stockholm, Sweden; 10 Department of Clinical Genetics, Uppsala University Hospital, Mid-Sweden Healthcare Region, Uppsala, Sweden; 11 Centre for Inherited Metabolic Diseases (CMMS), Karolinska University Hospital, Stockholm-Gotland Healthcare Region, Stockholm, Sweden; 12 Unit of Epidemiology, Institute of Environmental Medicine, Karolinska Institute, Stockholm, Sweden; 13 Department of Haematology, Oncology and Radiation Physics, Skåne University Hospital, Southern Healthcare Region, Lund, Sweden; 14 Division of Oncology, Department of Clinical Sciences Lund, Lund University, Lund, Sweden

**Keywords:** Health policy, Rare diseases, Registries, Health equity, Universal health care, Sweden

## Abstract

In the Swedish universal healthcare system, persons living with rare diseases (PLWRD) face major challenges in receiving diagnosis and accessing care,stemming in part from heterogeneity of disease and systemic barriers to care. This contributes to health inequity and a lack of visibility for PLWRD within thehealth system. This Perspective paper provides an overview of the facilitators and challenges during the development and implementation of a national raredisease (RD) quality registry, RaraSwed. The registry collects data on RD diagnosis. Adopting a social innovation and systems thinking methodology, inaddition to governance, have proven beneficial. RaraSwed supports RD research, evidence-based RD care, and Sweden’s National Quality Policy and Strategy(NQPS) for RD. Future modules on patient-reported and physician-reported data on care coordination, treatment and clinical attributes will capture the holisticpatient experience. RaraSwed may contribute to health equity for PLWRD in Sweden and in turn facilitate international RD data harmonisation.

## Background

According to the World Health Organisation (WHO), a national quality policy and strategy (NQPS) represents a country’s coordinated efforts to enhance the quality of healthcare across its health system [1]. The WHO Health Systems Framework identifies leadership/governance and health information systems (HIS) as building blocks for national health policy [2]. Developing and implementing a NQPS relies in part on the presence of clear and accurate performance data, which requires HIS for data collection, measurement, reporting, and the feedback loop necessary for improvement. Thus, HIS, such as National Quality Registries (NQR), are crucial in transforming health service delivery [3]. Health systems and services should be universally “effective, safe, people-centred, timely, equitable, integrated, and efficient” [1]. However, persons living with rare diseases (PLWRD) face complex multi-dimensional challenges that stem from both the inherent aspects of rare disease (RD) (e.g., low prevalence, heterogeneous presentation) and external barriers of fragmented global and national policies and strategies. These challenges exacerbate each other, leading to health systems worldwide failing to provide health equity to PLWRD [4].

The United Nations (UN) has urged all member states to accelerate their efforts to enhance the quality and equity of healthcare for the 263–446 million individuals living with RD, which include around 7.000 distinct conditions [5, 6]. This global policy priority is addressed through the UN 2030 Agenda for Sustainable Development [7], the UN Political Declaration on Universal Health Coverage [8], and the UN General Assembly’s adoption of the Resolution “Addressing the Challenges of Persons Living with a Rare Disease and their Families” in 2021 [9]. However, barriers to RD research are multifaceted, ranging from fragmented data and gaps in natural history to the inherent difficulty of comparing rare conditions with common ones [10, 11]. The scarcity of reliable data impedes the derivation of real-world evidence, making it difficult to formulate coordinated global and public health policies [10, 12–16]. NQRs are widely recognised as essential tools to address these challenges, facilitating healthcare planning, policy development, and the research of effective treatments for PLWRD [11, 15–17]. This paper outlines the concerted efforts in Sweden towards establishment, quality management and maintenance [17] of the Swedish NQR for RDs, RaraSwed. This strategic initiative aligns with Sweden’s broader health system strengthening [2, 18] strategy forming an integral component of the Swedish government’s emerging NQPS reform targeting RDs. We explore the pivotal facilitators and challenges addressed during the development of RaraSwed and consider its future in facilitating RD research. Ultimately, we aim to illustrate how robust data integration provides the necessary foundation for more equitable healthcare planning and policy development for RDs.

## Rare disease in Sweden and the national quality registry for rare diseases – RaraSwed

RDs are numerous, heterogeneous in nature, and specialist expertise is geographically scattered [4]. Research into RD global and public health has concentrated on proposing, establishing, and assessing national and multi-national quality policies and strategies [13, 19–22]. Although the approaches taken by various nations in terms of RD plans, policies, strategies, and legislation differ significantly, it has been proposed that countries within the European Union (EU) demonstrate the most unified legislative stance [21]. This includes adopting a common definition of RD and operating under the multi-national European Commission Orphan Medicinal Product Regulation No. 141/2000 [23]. However, the inherent low prevalence of RDs poses significant challenges in recognition and identification, including a diagnostic delay of several years on average [24]. Furthermore, the small sample sizes and heterogeneity of these diseases make it difficult to conduct high-value empirical research and derive standardised practices, guidelines, and policies of care [13]. As such, foundational knowledge on RDs remains limited in many countries. In Sweden, RD is defined as a condition affecting 1 in 10,000 inhabitants [25]. Recognised systemic limitations in providing cohesive care for PLWRD have been highlighted by both the Swedish government (Government Decision S2010/4935/HS) [26] and the national patient advocacy group, Rare Diseases Sweden [27]. A 2017 survey underscored these gaps, revealing that only 39% of patients received necessary medical care and 73% lacked vital rehabilitation. Consequently, the 2018 Government Bill (Motion 2018/19:2309) [25] called for a national action plan to ensure equity in health and social welfare for PLWRD in Sweden by optimising patient pathways and aligning financial support. To address this, the National System for Knowledge-Driven Management (NSKM) [18] established the Knowledge-Driven Management for Rare Diseases (KDM RD) in 2018. KDM RD, also known as National Programme Area of Rare Disease, aims to drive the development of national RD quality policy. It led the national strategic effort towards building a NQR of RD, RaraSwed, and remains the governing body responsible for the registry [28].

**Fig. 1:**
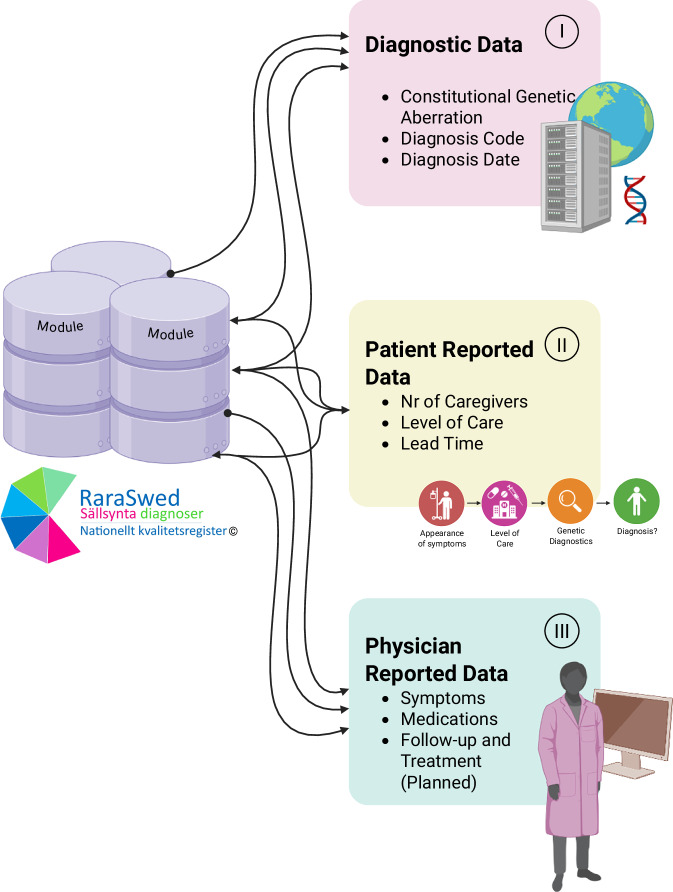
The Modules of RaraSwed. Figure illustrates an overview of three modules of RaraSwed. Module I (diagnostic) launched September 2023. Module II is underway and will collect patient-reported data. Module III is planned and will collect physician-reported data. RaraSwed logotype, www.csdsamverkan.se/halsoochsjukvard/raraswedkvalitetsregister/, used with permission.

The RaraSwed initiative was launched in August 2022. RaraSwed is a national, observational, and longitudinal registry. To establish its clinical foundation, the first diagnostic module was introduced in September 2023, aiming to collect data on the incidence, prevalence, diagnoses, and demographic profiles of PLWRD across 21 regions. Specifically, this module provides open data on the number of registered persons with genetically verified RD, corresponding ORPHA-codes [29] and gender ratios. To ensure transparency and accessibility, these data are published online at www.csdsamverkan.se/halsoochsjukvard/raraswedkvalitetsregister/. Furthermore, the data will be presented with other national and regional health registries to facilitate performance analysis through “Health Care in Numbers” [30], a service provided by SALAR. Detailed specifications for the complete minimum data set (list of variables) can be found in Supplementary Materials 1 [15, 31]. Building on the diagnostic capabilities of Module I, future development of RaraSwed will expand to collect real-world data, including patient-reported and physician-reported outcomes (Modules II and III, respectively, see Fig. 1). This expansion is particularly vital given the systemic barriers currently faced by PLWRD, due to the fragmented nature of primary care in Sweden [32], a single patient may see up to 40 different providers, making it difficult to capture the true burden of these conditions nationwide. Consequently, the upcoming Module II is designed to identify the strengths and weaknesses of the Swedish health system regarding care coordination [33]. In doing so, it aims to fulfil key objectives of the KDM RD [28] and Swedish public health policy (prop. 2017/18:249) [34]. Throughout this development, the national patient advocacy group, Rare Diseases Sweden [27], has been an integral partner in establishing Module I and remains involved in the ongoing creation of Module II and beyond. However, despite this collaborative framework, implementing a comprehensive RD NQR presents a unique set of complexities and challenges [35], which are discussed below.

## The national rare disease infrastructure of knowledge-driven management

**Fig. 2:**
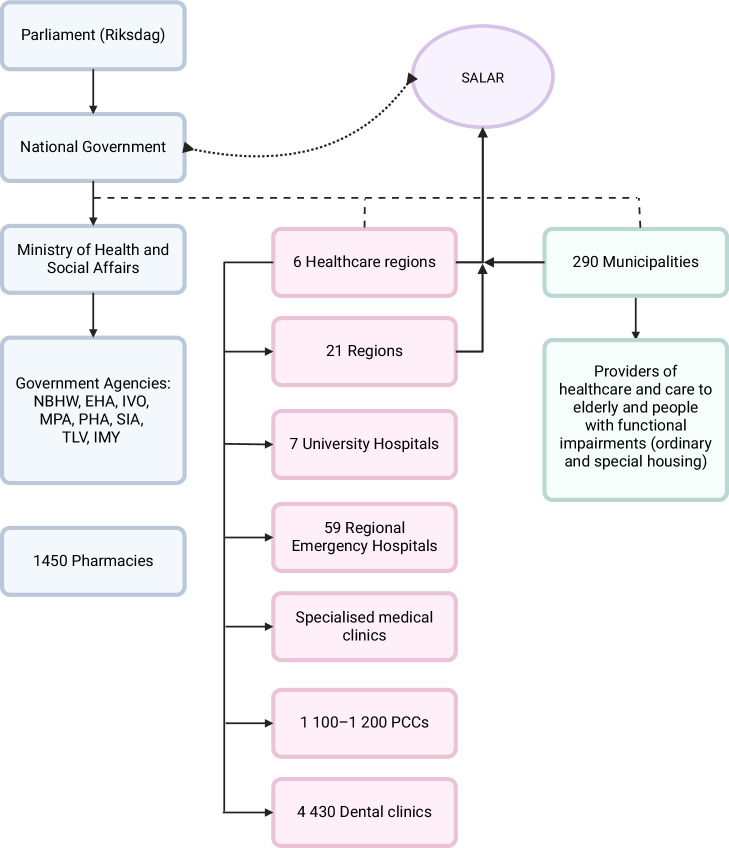
Overview of the Swedish Health System. Figure includes examples of government agencies. NBHW: The National Board of Health and Welfare / Socialstyrelsen; EHA: Swedish eHealth Agency (e-hälsomyndigheten); IVO: the Health and Care Inspectorate / Inspektionen för vård och omsorg; MPA: the Medical Products Agency / Läkemedelsverket; PHA: The Public Health Agency of Sweden / Folkhälsomyndigheten; SIA: the Swedish Social Insurance Agency / Försäkringskassan; TLV: the Dental and Pharmaceutical Benefits Agency / Tandvårds- och Läkemedelsförmånsverket; IMY: The Swedish Authority for Privacy Protection / Integritetsskyddsmyndigheten. PCC: primary care centre. SALAR: The Swedish Association of Local Authorities and Regions. Figure created by author, adapted from Janlöv, N., Blume, S., Glenngård, A. H., Hanspers, K., Anell, A., & Merkur, S. (2023). Health system review Sweden. In *Health Systems in Transition: Sweden* (Vol. 25, Issue 4). www.healthobservatory.eu

Sweden’s healthcare system is decentralised, overseen by the Ministry of Health and Social Affairs, managed by 21 autonomous regions and 290 municipalities, and primarily financed through taxation (see Fig. 2). To address regional disparities, the Swedish health system has implemented several reforms [32], focused on evidence-based guidelines and integrated clinical pathways. These collaborative efforts involve academic institutions, specialist groups, and community stakeholders to establish a cohesive, integrated health system. Significant developments include the 2017 standardisation of cancer care pathways (standardiserade vårdförlopp), and the 2018 concentration of highly specialised services (nationell högspecialiserad vård). Simultaneously, regional efforts such as the NSKM and primary care reinforcements have further supported this systemic integration [32].

**Fig. 3:**
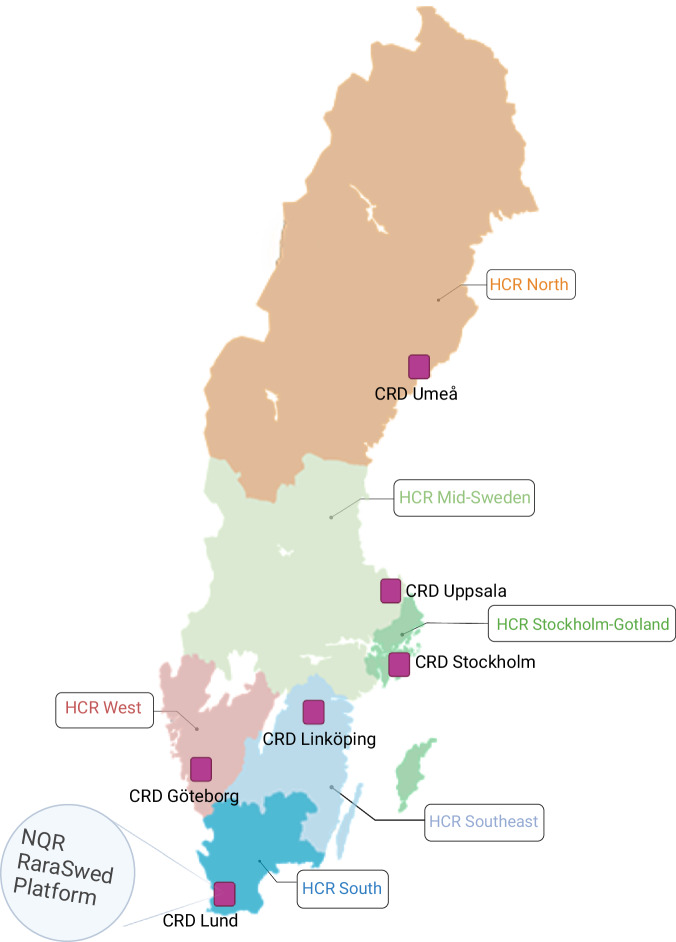
The Map of Sweden. Six healthcare regions (HCR) are visualised together with six Centre for Rare Diseases (CRD) nodes. The national quality registry of rare diseases, RaraSwed, platform is located in southern Sweden, collaborating with the southern CRD node. Figure adapted from Nationella kompetensrådet, regional samverkan, Socialstyrelsen, www.nationellavardkompetensradet.se/samverkan/regional-samverkan/, used with permission

**Fig. 4:**
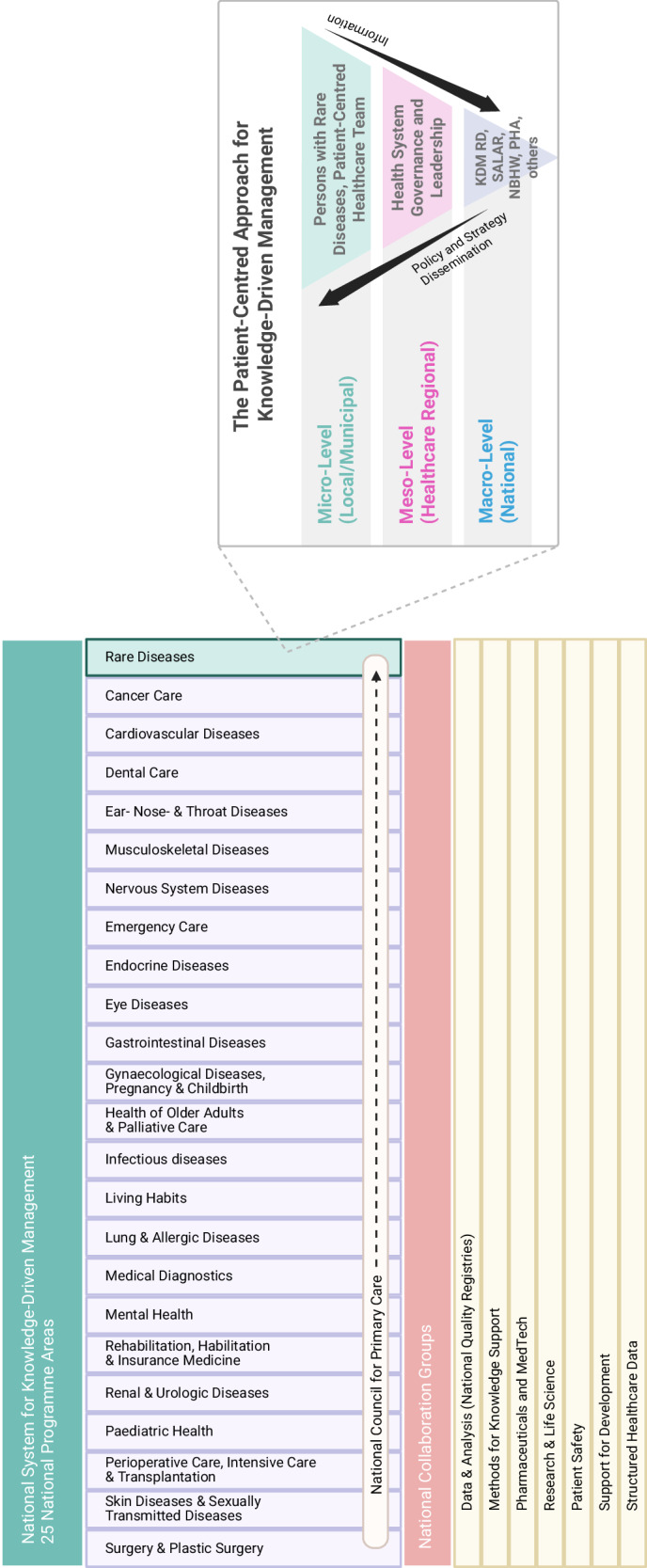
Overview of the National System for Knowledge-Driven Management Organisation in Sweden. The table depicts the 25 national programme areas and seven national collaboration groups. The pop-out figure illustrates information attainment, and policy and strategy dissemination. Figure adapted from Sjukvårdsregion Mellansverige, https://csdsamverkan.se/csdisverige/sjukvardsregionalacentrumforsallsyntadiagnoser/csd/csdsyd.5.5d8a5cd41793ff 87c4e585f.html, used with permission

The NSKM, since its establishment in 2018, is centred around collaborative development. It supports Sweden’s public health policy emphasising knowledge-driven management, decision-making and quality improvement [36]. The objective is to improve population health through high-quality care that is evidence-based, safe, person-centered, equitable, accessible, and efficient [18]. The system facilitates collaborative governance across the 21 autonomous regions, divided into six healthcare regions (HCR) centered around seven university hospitals which serve as regional hubs for research, education, and the funneling of care (see Fig. 3). It includes 25 national programme areas, and is modelled after the Confederation of Regional Cancer Centres, which, since its establishment in 2010, continues to coordinate cancer care in Sweden [37]. Each of the national programme areas of the NSKM focuses on a specific priority (see Fig. 4), such as RDs, and acts as an independent national operational unit. These infrastructures translate national public health policies and strategies into actionable measures to enable system-wide implementation and improve health service delivery across Sweden.

As part of NSKM, the KDM RD aims to: (1) improve early detection and management across all care levels; (2) foster stakeholder collaboration to integrate RD care; (3) reinforce the collection and integration of reliable RD data; and (4) promote RD awareness and educational training for professionals and the public [28]. These objectives are closely aligned with the ambitions of Sweden’s national quality policy (prop. 2017/18:249) [34, 36].

A fundamental tool for achieving a knowledge-based management in NSKM is the NQR, which serves to measure, evaluate, and improve healthcare operations, a prerequisite for clinical advancement. The foundation for this data-driven approach was established by the Population Registration Reform in the 1940s and the introduction of the Personal Identity Number [38], which enabled the development of national population registries and NQRs. Today, government-administered registries for patient care and mortality are overseen by the National Board of Health and Welfare [39], which also manages the Joint Action on Integrating the European Reference Networks into National Healthcare Systems, an initiative launched in March 2024 to facilitate the incorporation of these networks into the Swedish health system [40]. However, unlike these government-administered registries, NQRs are typically initiated by healthcare professionals and managed by registry steering committees [41], notably, they do not function as clinical decision support tools on the micro-level [42].

**Fig. 5:**
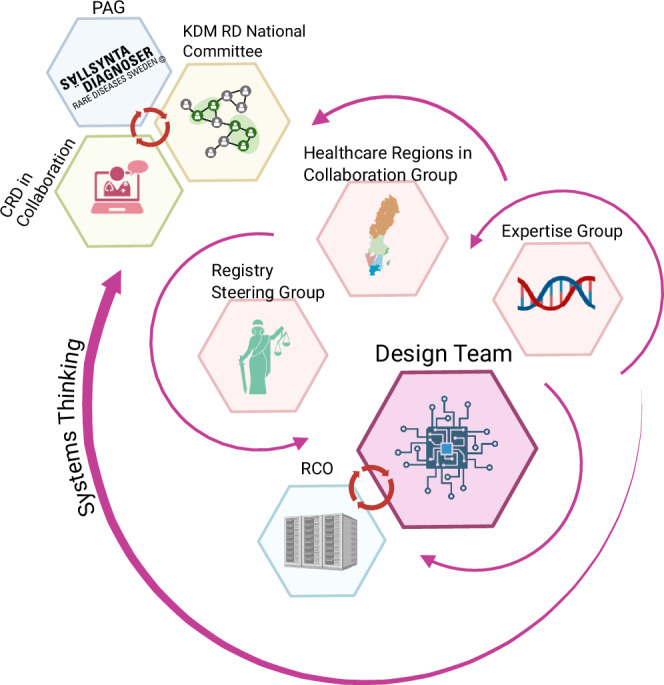
Governance of Knowledge-Driven Management of Rare Diseases, and Transparent Iterative Processes. Figure illustrating the multi-tiered organisational governance of knowledge-driven management of rare diseases. The systems thinking approach and transparent, iterative process of adapted design thinking method has been applied, incorporating continuous feedback from all stakeholders. Methodologies applied by the Design Team during the innovation and establishment process of RaraSwed. PAG: Patient Advocacy Group, KDM RD: Knowledge-Driven Management of Rare Diseases, RCO: Registry Centre Organisation, CRD in Collaboration: Centre for Rare Diseases in National Collaboration, Healthcare Regions in Collaboration Group. Sällsynta Diagnoser logotype; www.sallsyntadiagnoser.se/, used with permission

The governance of the KDM RD is organised into a multi-tiered structure to support operational needs (see Fig. 5). At the national level, a committee composed of representatives from all Swedish HCRs is responsible for policy work, while regional and local working groups translate this policy and coordinate with stakeholders, such as the Healthcare Regions in Collaboration Group. This ecosystem involves collaboration with patient organisations, industry, and government agencies like the Swedish Association of Local Authorities and Regions (SALAR). SALAR provides necessary funding to NSKM and KDM RD for the establishment of NQRs, such as RaraSwed. Furthermore, the KDM RD infrastructure integrates with six regional strategic operative units, Centres for Rare Diseases (Centrum för Sällsynta diagnoser, CSD) [43] nodes, established in each HCR (see Fig. 3) and connected to university hospitals to direct care across tertiary, specialist, or primary centres. The KDM RD governance and infrastructure were developed in parallel with the innovation and establishment of the RaraSwed registry.

Within each HCR, a Registry Centre Organisation (RCO) provides the necessary infrastructure and operational support for the NQRs [44]. Consequently, all NQRs are affiliated with an RCO and a Central Data Protection Authority [45], adhering to Swedish governance and data protection regulations, including the opt-out consent obligation [46]. However, despite this supportive infrastructure, Sweden’s decentralised governance and regional autonomy have resulted in a landscape where the six HCRs maintain independent electronic health record systems. This reliance on disparate systems can present significant challenges when leveraging health data for NQPS and may be a factor in the ongoing complexities of achieving national interoperability in Sweden.

## Building a national quality registry of rare diseases, RaraSwed

To overcome barriers of RD research, the standardisation of RD registries has emerged as a critical global priority to mitigate the challenges associated with data fragmentation and small patient populations. EU efforts have resulted in frameworks advising on and toward a systemic harmonisation of data elements and quality standards. Significant milestones include the 2012 European Organisation for Rare Diseases (EURORDIS – Rare Diseases Europe) [47] declarations and the 2017 launch of the European Rare Disease European Rare Disease Registry Infrastructure (ERDRI) [48], which established a “Set of Common Data Elements“ [49] to ensure cross-border interoperability. This strategy has been further reinforced by the European Joint Programme for Rare Diseases (EJP RD) [50] and the 2024 launch of the European Rare Diseases Research Alliance (ERDERA) [51] under the EU funded Horizon Europe framework [52]. The FAIR (Findable, Accessible, Interoperable, Reusable) guiding principles on registry implementation intend to enhance data reusability [53]. These collective efforts have established a robust RD foundation in Europe, aiming to ensure that national registries align with international data stewardship and quality standards.

The emerging patient registries in Europe vary greatly and may focus on diverse needs and outputs [54]. Some examples include registries on disease prevalence, incidence, and trends; physician-reported data on disease progression, treatments, and symptoms; drug or device data on efficacy and safety; and natural history based on patient-reported data [16, 55–57].

Sweden has more than 150 separate NQRs, that may include RD data [42]. Examples include organ-specific cancer registries, such as the Swedish National Quality Register for Brain and Central Nervous System Tumors [58], to population-specific cancer registries like the Swedish Childhood Cancer Register [59]. Others are disease-specific, including the Swedish Registry for Inherited Metabolic Diseases (RMMS) [60], and the Swedish Registry of Congenital Heart Disease (SWEDCON) [61]. However, these organ– and disease-specific NQRs primarily host granular micro-data rather than aggregated data sets. Consequently, it is not possible to extract generic RD data or obtain a holistic overview of the national RD landscape from these disease-specific NQRs alone. This lack of coordination means that registries often function as isolated data repositories where a comprehensive, systemic perspective is absent. While specific data may be requested from individual registries for research purposes according to national regulations, Swedish disease-specific NQRs generally do not enable data sharing between systems. Essentially, they function as closed, independent, and isolated data silos. For PLWRD, the problem of siloed data is particularly acute due to fragmented and scattered information across both national and international registries [62]. Building on this infrastructure, a comprehensive NQR for RDs covering all diagnoses across Sweden has been lacking until RaraSwed.

The RaraSwed initiative is one of the first nationally coordinated strategies for RDs developed through collaborative policy discussions. The Swedish government’s efforts from 2018 to 2020 (Government Decision S2017/05457/FS, prop. 2017/18:40) [63, 64] catalysed this national health system strengthening effort to address the country’s RD health planning needs. The KDM RD national committee has been actively involved in NQPS discussions and infrastructure implementation to drive this progress, as outlined in the 2020 situational analysis [65].

Given the dispersed nature of PLWRD within the Swedish healthcare system, our initial focus was on devising a strategy to capture these individuals comprehensively and geographically inclusively. Despite their presence throughout healthcare, PLWRD constitute a heterogeneous group, exhibiting varying degrees of symptoms of disease. Consequently, considering that 80% of individuals with RD have a genetic abberration [66], a key solution was to integrate the capture of these individuals via Clinical Genetics clinics, thereby ensuring comprehensive inclusion without selection bias. Clinical Genetics clinics, centralised in Sweden with seven total units located at each university hospital, became central to this effort. The regional centers for rare diseases, in close proximity, provided crucial support to this work. However, RaraSwed does not compete with existing, more targeted registries, it serves as a bridge connecting and enhancing the overall RD data landscape in Sweden. Ultimately, this registry addresses the foundational knowledge gap on RDs in Sweden and will be Sweden’s contribution to the broader European RD data and research platform.

To support the operationalisation of this strategy, RaraSwed is overseen by a national steering group comprised of representatives from all six HCRs with expertise in RDs. Region Skåne, the southern HCR in Sweden (see Fig. 3), is designated the Central Data Protection Authority for RaraSwed. The RCO South provides the necessary technical infrastructure and facilitates discussions on the user interface. Funding from SALAR and the KDM RD, as well as a dedicated grant, has supported the development of the first diagnostic module of RaraSwed.

## RaraSwed modified design method

The development of RaraSwed was led by the Design Team, which serves as the on-site practical operating unit tasked by the KDM RD. To develop, establish, and implement RaraSwed, the team operates in close collaboration with the RCO, the registry steering group, expertise group, and the Healthcare Regions in Collaboration Group (see Fig. 5). The team is headed by a project manager, also known as registry holder. The registry holder aligns the RaraSwed operational framework vision with the emerging national RD-, and existing public health quality policy [28, 36], anchoring it within the KDM RD infrastructures. The Design Team process leader is responsible for the operational implementation and interface design of RaraSwed.

The establishment of RaraSwed has been facilitated through a systems thinking approach [67] and the application of social innovation in health, including the emerging human-centred design methodology and design thinking [68]. Systems thinking involves understanding the connections, relationships, and synergies among the parts of the whole system, with applications in both health systems quality and public and global health [69, 70]. Social innovation emphasises “bottom-up” community-engaged processes, as community-driven programmes can lead to more sustainable and accountable solutions, with the potential to improve population-level health. Design thinking is an iterative, user-centric problem-solving approach that integrates community needs and perspectives, incorporating continuous feedback into the final product [68]. The RaraSwed Design Team approach is based on the five stages of the Stanford d.School Design Thinking Framework: Empathise, Define, Ideate, Prototype, and Test [71], adapted by the process leader for the specific RaraSwed context.

**Fig. 6:**
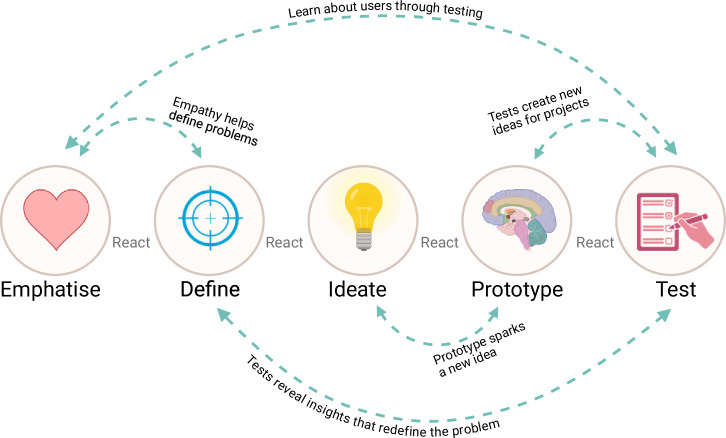
Design Thinking: a Non-Linear Process. Figure illustrates the modified design thinking approach by Design Team, reflecting the non-linear nature of design thinking described by Interaction Design. Figure adapted from Interaction Design Foundation, The 5 Stages of Design Thinking: www.interaction-design.org/literature/article/5-stages-in-the-design-thinking-process/, used with permission

The core Stanford d.School Design Thinking Framework remained unchanged (see Fig. 6), with the addition of a “React” stage to incorporate feedback between each step. The Design Team employed systems thinking methodology throughout the iterative design thinking process, engaging in open and transparent dialogues with the diverse stakeholders within the KDM RD (see Fig. 5). Furthermore, reflecting the non-linear nature of design thinking described by Interaction Design [72] the process leader and RaraSwed Design Team navigated flexibly between stages in response to emerging insights and stakeholder input. The project was structured into iterative “Sprints”, with a workbook-method tracking insights, changes, and resources.

For more comprehensive information on the design thinking methodology application in the RaraSwed initiative, please refer to Supplementary Materials 2, which include workshop examples of iterative processes, an excerpt from the workbook, and the project timeline with detailed explanations of the sprints.

### Key facilitators and challenges during the development, establishment, and implementation of KDM RD and RaraSwed

The research group highlights that a strong NSKM foundation with government funding is key to the successful establishment of a NQR for RD in Sweden [14, 31, 73, 74]. However, the evolving KDM RD infrastructure and incomplete governance posed challenges in aligning the RaraSwed platform while policies were simultaneously being developed. Central to addressing these challenges was the implementation of systems thinking and design thinking approaches by an experienced Design Team. This, along with the network provided by the NSKM, drove the project to consensus.

The inherent and external challenges of RDs are outlined below, along with considerations for addressing them through RaraSwed and the KDM RD.

### Challenges of extracting reliable RD data

Non-standardised coding practices for RDs present a significant challenge for developing HIS for RDs [75]. Several hospitals and registries use coding frameworks such as the International Classification of Diseases Tenth Revision, (ICD-10) [76], which is mandatory for diagnostic reporting within the Swedish healthcare system. Meanwhile, the Systematised Nomenclature of Medicine Clinical Terms (SNOMED CT) [77], has been implemented to varying extents. These frameworks often lack the specificity to capture RDs accurately, e.g., clinical practice mainly relies on ICD-10 in Sweden. This reliance makes PLWRD invisible in the healthcare system and contribute to health inequities [12]. To address this, international efforts are integrating standardised coding systems, including ORPHAcodes, the International Classification of Diseases 11th Revision (ICD-11), Online Mendelian Inheritance in Man (OMIM), and Human Phenotype Ontology (HPO) into RD registries [20, 29, 78–85]. RaraSwed has similarly incorporated these coding systems to improve visibility. While not intended for direct clinical care of individual patients, RaraSwed aims to reduce diagnostic delays and support targeted quality improvement initiatives for PLWRD at a group level [28]. This further supports the nationwide implementation of RD coding systems capturing high integrity, aggregated data in Sweden.

Globally RDs are underreported, underrecognised, and underfunded [82]. Development of registries like RaraSwed allows for harmonised data collection, providing insights into disease progression and helping to identify and address knowledge gaps [86]. To ensure reliable RD data, quality evaluation studies of RD registries highlight the need for high data integrity and validity [10, 73–75]. The involvement of a patient advocacy group, such as Rare Diseases Sweden [27], is also considered important in generating valid data [14, 87]. It is further argued that considerations of quality management, sustainability, and maintenance should be incorporated into the design of RD registries [16]. RaraSwed ensures reliable, high quality data and quality maintenance by training data stewards and using standardised data collection, which reduces entry errors for accurate input [14, 16, 35]. Regular data steward check-ins for feedback, along with RCO collaboration, have been a key facilitating factor in achieving high data integrity [73]. Maintaining quality while integrating real-world data will help derive reliable data contributing to real-world evidence, ultimately enhancing the validity of the collected data [10]. While RaraSwed focuses on genetically verified cases, it aims to increase genetic testing and provider education to capture undiagnosed cases.

### Challenges in harmonising RD data transition from local to global level

Achieving interoperability is a challenge but crucial for coordinating evidence-based RD care through efficient data sharing and integration [10, 11, 74, 75]. A key strength of RaraSwed is the centralised collection of diagnostic genetic data, captured through genetic testing performed at any of the seven Swedish Clinical Genetics clinics. The comprehensive RD data spanning all of Sweden, is a valuable example of achieving harmonised RD data while maintaining data integrity, reliability, and validity. The geo-mapping function may further aid systematic comparison of HCR care coordination performance for PLWRD, guiding targeted interventions, after the launch of module II.

RD registries across Europe are considering interoperability [86, 88] by adopting EU standards [47, 48, 50, 51, 89] and FAIR principles [53] – essential for managing big data healthcare complexities [17, 90]. Once fully established, RaraSwed aims to align with these frameworks and will capture enriched multidimensional integrated data, according to the previous existing recommendations on a national RD registry [17, 31, 35, 73]. However, as previously noted, Sweden faces interoperability challenges due to disparate electronic health record systems. Consequently, while NQRs like RaraSwed endeavor to implement FAIR principles, this does not automatically translate to achieving FAIR standards at a national big data level. This underscores the ongoing necessity to address interoperability barriers within the Swedish healthcare infrastructure.

### Aligning directions and achieving consensus during implementation

To the best of our knowledge, our work is the first to describe a systems thinking approach and social innovation, such as Design Thinking, in RD health and policy planning, and registry development. This methodology combination has been key to overcoming the challenge of aligning a decentralised healthcare system towards shared goals, and the inherent and external barriers of RD research. While establishing aligned national infrastructures is daunting, we have learned it is achievable, though it may require compromise. Our findings demonstrate the benefits of using social innovation and systems thinking methodologies in global health, which distinguishes our work in the field of RD research [70]. Sweden’s decentralised governance poses its own set of challenges. We believe a cultural shift towards integrative systems thinking among healthcare organisations and professionals, overcoming power dynamics and inertia, is essential [68, 69, 91]. NSKM is an important cornerstone in overcoming these challenges, especially through the use of NQRs. Furthermore, the social and cultural context of the population influences benchmarking and the need for culturally appropriate indicators [92].

The successful innovation, establishment, and implementation of a NQR RD requires a delicate balance between data standardisation, interoperability, governance, stakeholder engagement and quality assurance. The RaraSwed initiative aligns with the WHO health systems strengthening approach, aiming to improve the HIS and governance/leadership building blocks to achieve more equitable and lasting improvements in health service delivery for PLWRD. Our methodologies and insights can inform policymakers and assist other countries in developing RD NQPS, health system strengthening, and national registry platforms. This can also apply to quality development and improvement of a healthcare system in general.

## Conclusions

Existing governance, like NSKM, and methodologies like social innovation and systems thinking have been fundamental in the development of RaraSwed. This NQR RD will be a resource that supports patient care, research, and policymaking in the field of RDs. It serves as a valuable tool for extracting homogenous data from heterogenous diseases, to drive forward the NQPS on RDs in Sweden, anchored in real world data and natural history of PLWRD.

The Swedish government has directed the National Board of Health and Welfare to develop a national strategy for RDs (Government Decision S2024/00038) [93], in which RaraSwed forms an integral part. Additionally, the government has tasked the Dental and Pharmaceutical Benefits Agency with analysing conditions and devising tools to enhance access to orphan drugs within Sweden (Government Decision S2024/00481) [94].

Furthermore, a primary care reform (Government Decision S2022/04844) [95], is currently being developed and implemented, with the objective of enhancing care coordination and patient-centred care through primary care system strengthening initiatives. These reforms aim to improve access to, and the quality of, primary care, as well as to better integrate primary care with specialist and tertiary services. The final outcomes of the reform in terms of the RaraSwed RD data and its role in coordinating care for PLWRD will need to be evaluated.

The vision of harmonising and integrating RD data across Europe is promising. Reliable data and real-world evidence can guide health policies for RDs. By providing cohesive access to care and unifying collective efforts, we can elevate the standards of care, improve care coordination, and mitigate the barriers faced by PLWRD worldwide.

For further core terminology used throughout this work, please refer to glossary of terms in Supplementary Materials 3.

## Supplementary information

Supplementary Materials 1 Title: Minimum Data Set, Variable list, RaraSwed. Description: This document provides an excerpt of the RaraSwed variable list in Swedish. It details the Minimum Data Set and structural framework utilised within the database to ensure standardised data collection

Supplementary Materials 2 Title:Dan Hellström Modified Design Thinking Methodology. Description: This material provides a comprehensive description of the Dan Hellström Modified Design Thinking Methodology as applied during the innovation, establishment, and implementation of RaraSwed. It illustrates practical examples from each stage of the design process, including the application of the Workbook method and the use of Sprints to facilitate iterative development

Supplementary Materials 3 Title: Glossary of Terms. Description: This glossary serves as a reference for the core terminology used throughout this work and is divided into two distinct sections. The first section contains a translation guide providing English-to-Swedish translations for key health organisation and administrative terms, while the second section offers a comprehensive health system strengthening glossary adapted from the World Health Organization Glossary List to provide necessary context for the systemic aspects of the RaraSwed implementation

## Data Availability

Not applicable.

## List of Abbreviations

HCR: Healthcare Region
HIS: Health Information System
KDM RD: Knowledge-Driven Management for Rare Disease
NSKM: National System for Knowledge-Driven Management
NQPS: National Quality Policy and Strategy
NQR: National Quality Registry
PLWRD: People living with rare disease
RCO: Register Centre Organisation
RD: Rare Disease
