# Supplementary material for: Extracting homogenous data from heterogenous diseases: RaraSwed, the Swedish national rare disease quality registry
Source: BMC Glob Public Health. 2026 Jun 16;4:58. doi: 10.1186/s44263-026-00276-9 (PMC13270570; doi:10.1186/s44263-026-00276-9)
Supplement: Supplementary file 1 — Supplementary Materials 1 Title: Minimum Data Set, Variable list, RaraSwed. Description: This document provides an excerpt of the RaraSwed variable list in Swedish. It details the Minimum Data Set and structural framework utilised within the database to ensure standardised data collection [file 44263_2026_276_MOESM1_ESM.pdf]

Supplementary Materials 1: Minimum Data Set, Variable list, RaraSwed

Variable ID	Variable Name	Variable Name (Swedish)	Form variable text (Swedish)
105240	Social Security Number	Personnummer	Personnummer*
106026	Referring healthcare unit	Remitterande_vardenhet	Remitterande vårdenhet
106027	Referring clinic	Remitterande_klinik	Remitterande klinik
103882	Genetically verified pathogenic variant	Genetisk_bekraftad	Har patienten genetiskt bekräftad patogen variant?*
103884	Date of analysis / Analysis date	Datum_analyssvar	Datum för analyssvar?*
103885	Variant?	Variant	Variant:*
103902	Allele 1 klassification?	Klass_allel1	Klassifikation:*
103903	Denovo allele 1 klassification	Denovo_allel1	Bedöms förändningen vara de novo:*
103886	Mode of inheritance / pattern of inheritance	Nedarvd	Nedärvningsgång:*
104318	Autosomal recessive mode of inheritance	NedarvdTyp	Autosomalt recessivt:*
103896	Gene and chromosomal abnormality	gen_kromosomavvikelse	Gen:*
103898	NM	NM	NM:*
**Drop-down menu opens depending on the variant chosen**			
103899	Sequence variant	sek_snv_1	Ange sekvensavvikelse:*
103900	Genomic position of sequence variant	position_snv_1	Ange genomisk position:
104323	Gene of clinical significance	Gen_strukturell_2	Gen av klinisk betydelse:
104325	Structural variant / structural abnormality	sek_strukturell	Ange strukturell avvikelse:*
104745	DNA methylation	DNAmetylering	DNA-metylering
104746	Epigenetic?	Epi_vetej	Vet ej
104754	Maternal allele	metyl_mat_epi	Maternell allel
104756	Paternal allele	metyl_pat_epi	Paternell allel
104749	Heteroplasmy	het_plasmi	Heteroplasmi
104752	Homoplasmy	hom_plasmi	Homoplasmi
104782	Blood	Blod	Blod
104783	Muscel	Muskel	Muskel
104784	Urinary sediment	Urin	Urinsediment
104785	Buccal swab	Bucca	Buccaskrap
104786	Fibroblasts	Fibro	Fibrolaster
104787	Degree of heteroplasmy	Hetroplasmigrad_annat	Annat
104768	Isodisomy (Uniparental Isodisomy)	upid	Isodisomi (Upid)
104769	Heterodisomy (Uniparental Heterodisomy)	uphd	Heterodisomi (Uphd)
104788	Maternal uniparental disomy (UPD)	Mat_UPD	Maternell allel
104789	Paternal uniparental disomy (UPD)	Pat_UPD	Paternell allel
104320	Sequence variant	sekvensav_avvikelse_2	Ange avvikelse:
104321	Allele 2 klassification?	Klass_allel2	Klassifikation:*
104322	Denovo allele 2 klassification	Denovo_allel2	Bedöms förändringen vara de novo:*
104771	Analysis method	Analysmetod	Vilken analysmetod användes:*
104776	Number of analysed genes	Antal_analyserade_gener	Vid panel hur många gener har analyserats:
104794	Single/Trio analysis	Singel_trio	Singel/Trio analys:
105022	OMIM*	OMIM_stjarna	Ange OMIM* för gen:*
105023	Clinical symptoms indicative of pathogenic vari	Klinisk_symptom	Har patienten klinisk symptom kopplat till sjukdomsorsakande variant:*
105026	ORPHA	orpha	Ange ORPHA:
105027	OMIM#	OMIM_hashtag	Ange OMIM# för fenotypisk beskrivning:
105032	ICD-10 Code	icd10	ICD-10
105033	ICD-11 Code	icd11	ICD-11
105044	Genetic counselling	Genetisk_vagledning	Har patient/vårdnadshavare erhållit genetisk vägledning:
105161	Primary care	primarvardPre	Primärvården
105163	Specialist care	specialistvardPre	Specialistvården
105165	Clinical Genetics	kliniskGenetikPre	Klinisk Genetik
105169	Date less year?	datumPre	Datum (Månad − År)
105162	Primary care	primarvardPost	Primärvården
105164	Specialist care	specialistvardPost	Specialistvården
105166	Clinical Genetics	kliniskGenetikPost	Klinisk Genetik
105170	Date less year?	datumPost	Datum (Månad − År)
106211	Comment field / Remarks section	kommentar	Kommentarsfält:
109777	Incompleted registration	Ej_avslutad_registrering	Kryssa i box
105035	HPO	HPO1	Ange HPO:
