# Supplementary material for: Extracting homogenous data from heterogenous diseases: RaraSwed, the Swedish national rare disease quality registry
Source: BMC Glob Public Health. 2026 Jun 16;4:58. doi: 10.1186/s44263-026-00276-9 (PMC13270570; doi:10.1186/s44263-026-00276-9)
Supplement: Supplementary file 2 — Supplementary Materials 2 Title:Dan Hellström Modified Design Thinking Methodology. Description: This material provides a comprehensive description of the Dan Hellström Modified Design Thinking Methodology as applied during the innovation, establishment, and implementation of RaraSwed. It illustrates practical examples from each stage of the design process, including the application of the Workbook method and the use of Sprints to facilitate iterative development [file 44263_2026_276_MOESM2_ESM.pdf]

Supplementary Materials 2: Dan Hellström Modified Design Thinking Methodology

Method Description: Use of Social Innovation in the Development of RaraSwed

This section describes the systematic approach that the Design Team process leader used to apply various social innovation methods, such as Design Thinking, during the development of RaraSwed. To enable a structured and flexible design method process, several design methods have been integrated into distinct stages, adapted to the specific challenges and needs of each stage. Tasks present different degrees of challenges and concepts. The participants involved in the tasks, as well as the users and stakeholders have varying levels of experience with quality registries and design thinking. This has influenced the choice of methods and adjustments to method models in specific tasks.

Before starting the role as a process leader, three relatively quick and rough tasks were carried out to structure and organise the future work.

1. Strategic Direction

Establish an overarching primary approach and strategic direction in execution. By defining this direction early, the process leader ensured a consistent workflow and created a structure to facilitate the project's traceability and understanding. The strategy encompassed both the selection of primary design methods and overall goals for the visual and functional experience.

2. Organisational Analysis and Stakeholder Mapping

After determining the project's work methodology, we focused on analysing and mapping the organisational structure and identifying governance and stakeholders. This involved clarifying the project's governance through ownership, steering group, and responsibility allocation among the involved stakeholders. By creating a clear organisational overview, we ensured that each part of the project could be anchored with relevant stakeholders and that communication pathway between different parties was clear and concise.

3. Analysis and Contextual Understanding

With an established organizational structure, we began an overview analysis to investigate existing products and services in the same category. The purpose of this analysis was to create a current state picture, enabling us to identify key concepts, functional choices, and areas of use. This not only provided a reference framework for understanding current design norms and user needs but also a foundation for questioning existing structures and identifying opportunities for innovation.

Summary of the Initial Steps in the Design Process

The described initiation aims to enable a quick and effective start to the work. If an existing structure and documentation are available, the process can be shortened by integrating this information into the current work. The following steps summarise the initiation of work:

1. Establish methodology and strategy – Choose and formulate the methodological strategy that will form the basis of the design process. A primary approach for our work was visual communication and the workbook method. (See the section on choosing the primary approach).
2. Map organisational roles – Create a rough structure for the organisation, including governance responsibility allocation in between stakeholders, steering group, project owner, and stakeholders (an illustrative address book). (Figure 1.)
3. Conduct market analysis – Analyse existing services, their purposes, and relevant concepts to facilitate both design choices and the development of the project's innovation potential.

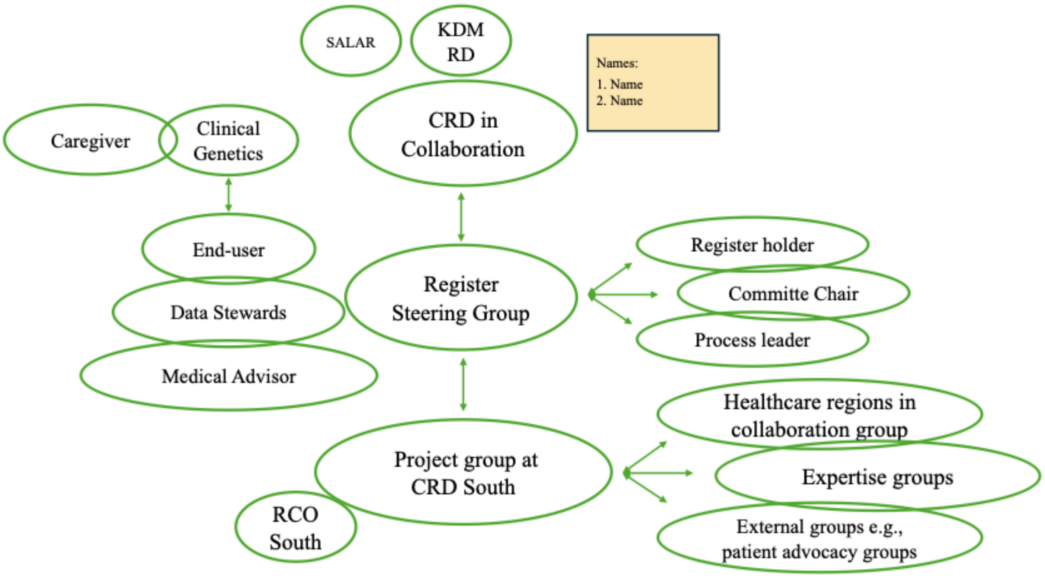

Figure S1. Organisational Chart during RaraSwed

Choice of Primary Approach in the Design Process
During the development of a national quality registry, two main approaches were chosen to support the design thinking process: visual communication and the workbook method. Both methods help the process leader improve understanding, overview, and collaboration within the different workgroups, while also facilitating documentation and reflection. Below, the purpose and advantages of the methods are presented.

Visual Communication as the Primary Approach
Visual communication serves as a tool to facilitate understanding and increase engagement in the design thinking process (Figure 2). By continuously using images, sketches, and prototypes during meetings and workshops, a clear reference point is created that participants can reflect upon and provide feedback on. This visual approach contributes to more efficient communication and strengthens the interaction between the steering group and users.

Purpose and justification:
- Improved interaction and alignment: Visual representations such as sketches and prototypes facilitate a more engaged and interactive collaboration. Visual aids can improve mutual understanding by providing a common reference point, which creates opportunities for deeper dialogue and a more user-centred approach.
- Support for national working groups: By visualising ideas and concepts in simple formats, it becomes easier for the working groups to follow the process's progress and make decisions. Visual communication contributes to structured follow-up, transparency and reduces the risk of misunderstandings.
- Quick overview and system understanding: A visual presentation of complex systems helps both users and the steering group understand various aspects of the product. In information design, visual aids can streamline the implementation process and minimise cultural differences and misunderstandings.
- Cost-effectiveness and flexibility: Using sketches and rough prototypes enables cost-effective testing and iterations. By visualising ideas early, before significant investments have been made into the project, different solutions can be explored freely and adjustments are made easy according to the feedback received. This method supports the flexible and iterative nature of the design thinking process, which is central to maximising resource efficiency in development projects.

The Workbook Method for Documentation and Reflection
The workbook method involves brief daily notes about final decisions, insights, and challenges (Figure 3). This continuous documentation functions both as a logbook and a reflection tool, which can drive the work towards goals and improve traceability in the process.
Purpose and justification:

- Promotes reflection and awareness of challenges: By using the workbook to document brief insights and reflections from each day or meeting, the process leader/group can reflect on progress and identify potential obstacles. Reflection strengthens learning and improves problem-solving skills, which is highly beneficial for managing complex design challenges.
- Creates new insights and supports decision-making: Documentation serves as a tool for generating new insights, leading to better understanding of decisions made and their impact on end-stage-product and its utilisation. By having access to notes from previous decisions and reflections, the process leader/group can make rational decisions.
- Improved traceability: The workbook improves traceability by creating a clear documentation history of the various steps in the design thinking process. This is particularly valuable in an iterative development environment where ideas and solutions are constantly reassessed. Traceability also increases transparency and ensures that critical decisions can be reviewed later, which is essential for quality assurance and follow-up.
- Keeps focus on goals and purpose: Design thinking processes are often iterative and can result in many ideas and solutions. The workbook serves as a reference point to help stay focused on the project's overarching goals and purpose. Structured documentation reduces the risk of losing direction and ensures that the project remains goal-oriented and user-centred.

Summary

Both visual communication and the workbook method were key components in the design thinking process and the project development work. Visual communication improves collaboration and understanding of complex ideas through images and sketches, which helps stakeholders participate in decision-making and follow-up. The workbook method, on the other hand, ensures structured and continuous documentation of reflections and decisions, improving traceability and keeping the project focused. Together, these approaches offer a balanced method for handling both creative and practical challenges in an iterative design environment.

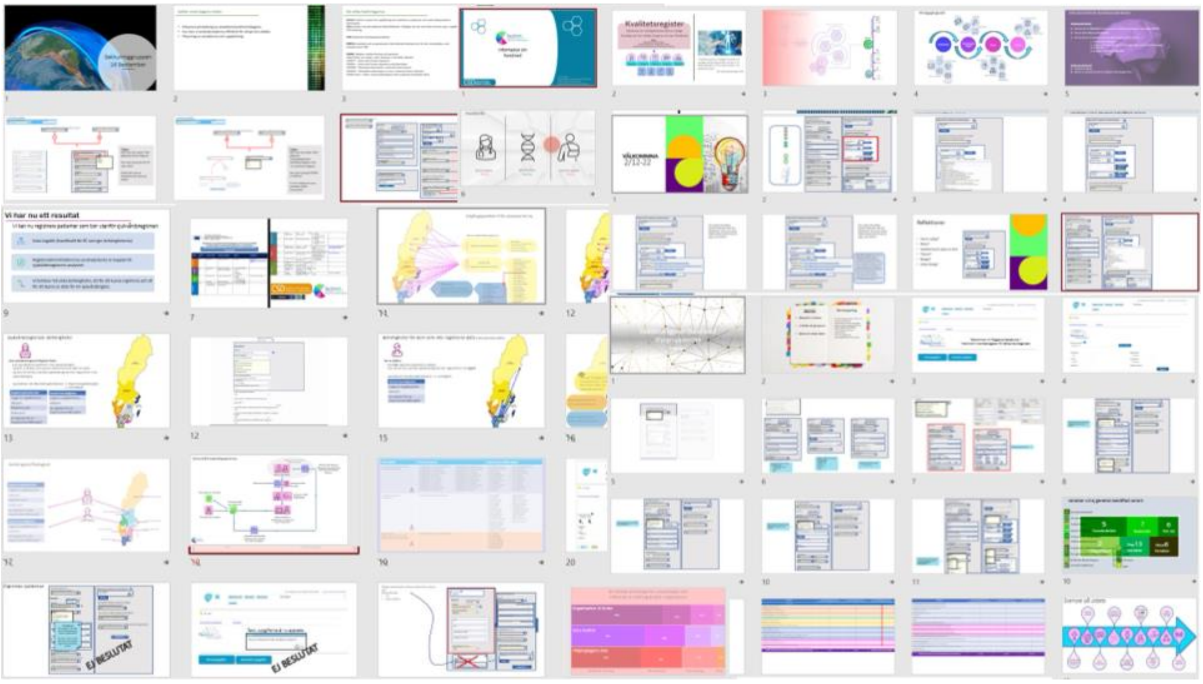

Figure S2. Visual Communication Chart during RaraSwed

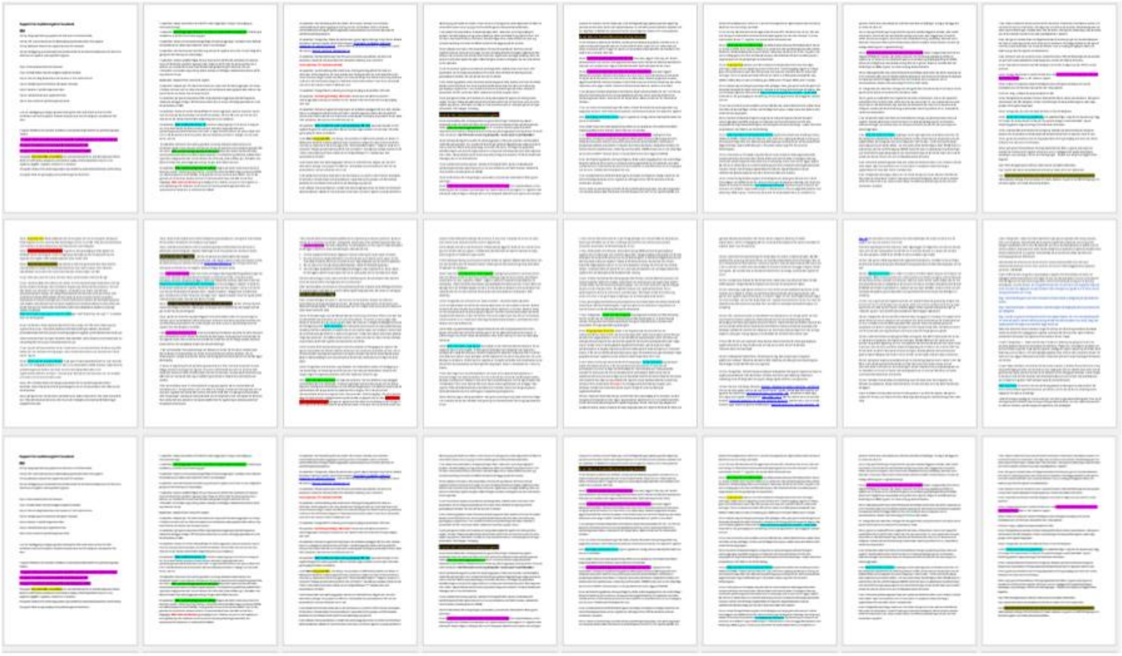

Figure S3. Overview of Excerpts from Dan Hellström Workbook

Design Process for National Quality Registry: A Manual

Developing a national quality registry (NQR) for rare diseases (RD) has involved navigating a complex context of legal statutes, cultural differences, and variations in current healthcare IT infrastructures. A structured and iterative work methodology was considered beneficial to create a shared understanding of needs and challenges. Design Thinking (DT) was chosen as the method, as its five steps—Empathy, Define, Ideate, Prototype, and Test—offer a flexible, iterative process that facilitates problem redefinition and the exploration of creative solutions from a user-centred approach. (Figure 4.)

Below is a description of the various stages in the process and the method tools used to develop the quality registry.

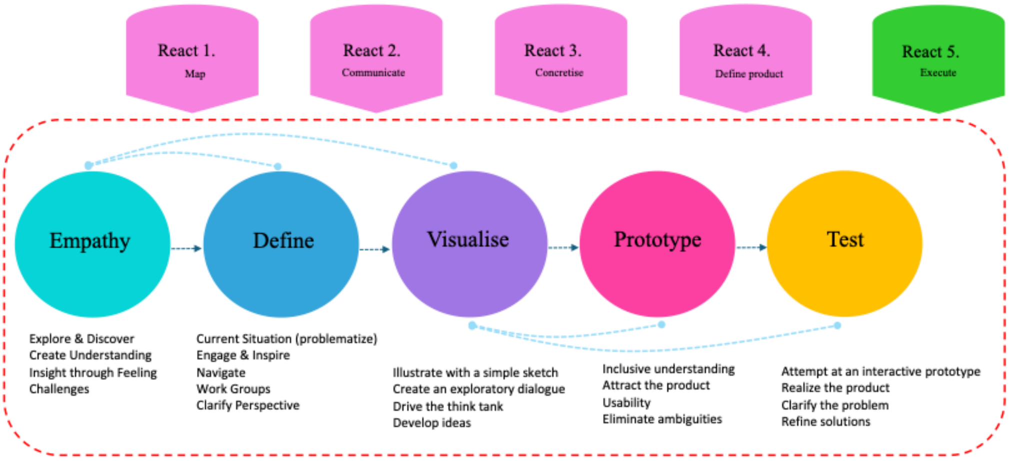

Figure S4: Modified Design Thinking Method Process during RaraSwed

Step 1: Empathy

The empathy phase aims to create a deep understanding of the user's perspective, needs, and values. We focused on listening and mapping the user's experiences through dialogues and observations. This user-centred approach involved building trust and creating an open dialogue to gain insights into the real needs.

Methods:

- Active listening and dialogues: By adopting a "beginner's attitude" and avoiding passing judgment early in the process or analysis, we created an atmosphere for open and free conversations.
- Visualization of insights: After each meeting, observations were documented, and sketches were made to clearly illustrate the user's wishes and needs.
- Simple visualizations: We used mind maps and simplified process charts to organise our insights. These tools helped structure the dialogue and engage participants by clarifying discussions. (Figure 5.)

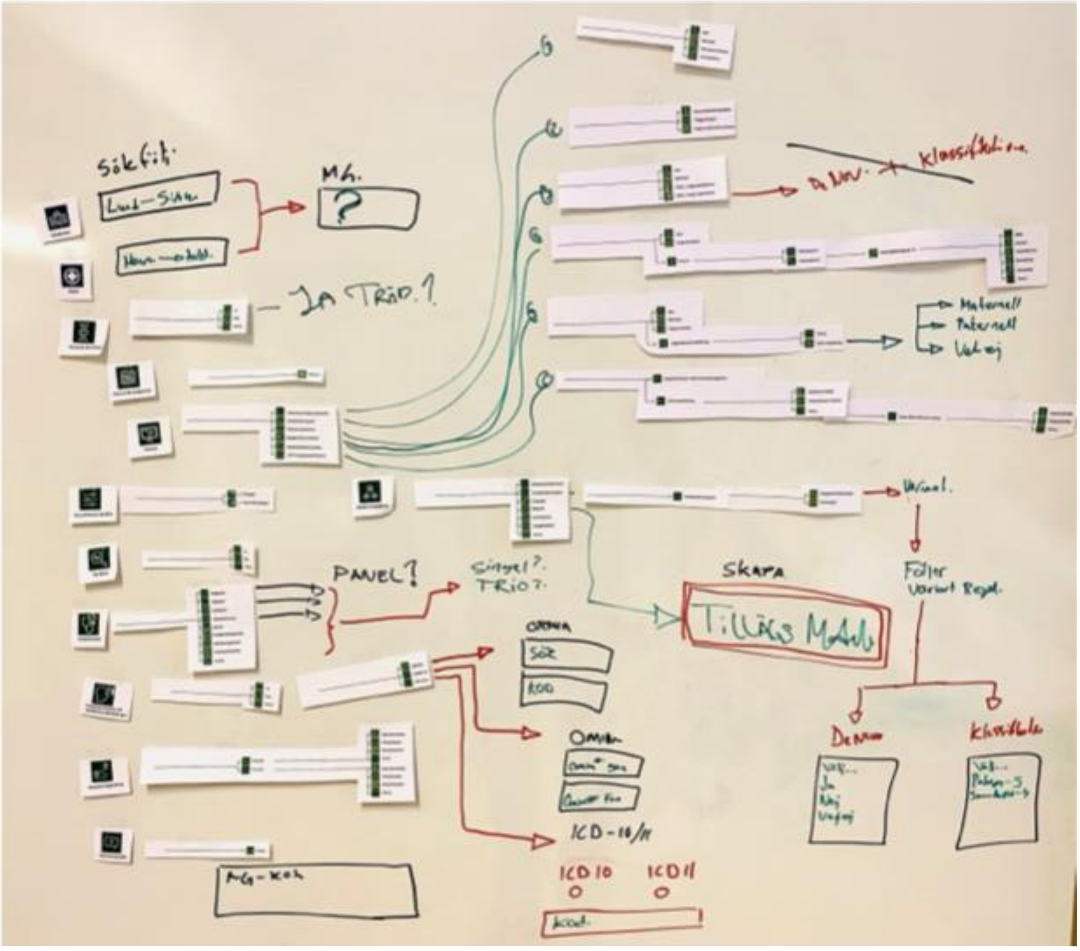

Figure S5A. Photograph of Whiteboard during Empathy Phase.

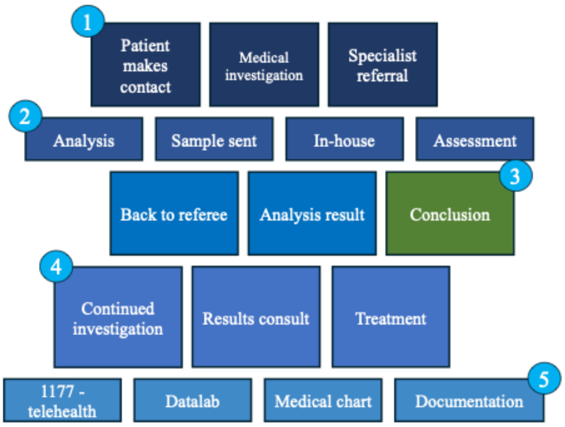

Figure S5B. Visualisation Chart during Empathy Phase.

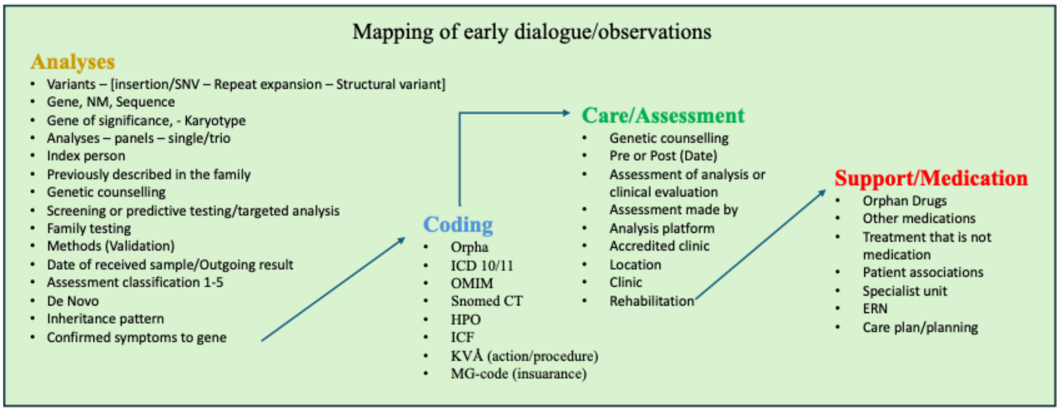

Figure S5C. Mapping of Early Dialogues and Observations

Step 2: Define

In the defining phase, empathy insights are transformed into a simple and clear problem statement, a "point of view" (POV). The goal is to clarify the core problem and identify specific user needs that can serve as a reference frame for further development.

Methods:

- Compilation and grouping of needs: By grouping and structuring the needs and experiences identified during the empathy phase, we got a clearer picture of the users' specific problems.
- "How might we" questions: These questions were formulated to transform insights into design challenges that inspire solutions and innovation.

- Reflection compilation: Between meetings, collected reflections were structured, which helped turn insights into conceptual pieces of information that could drive design solutions. (Figure 6.)

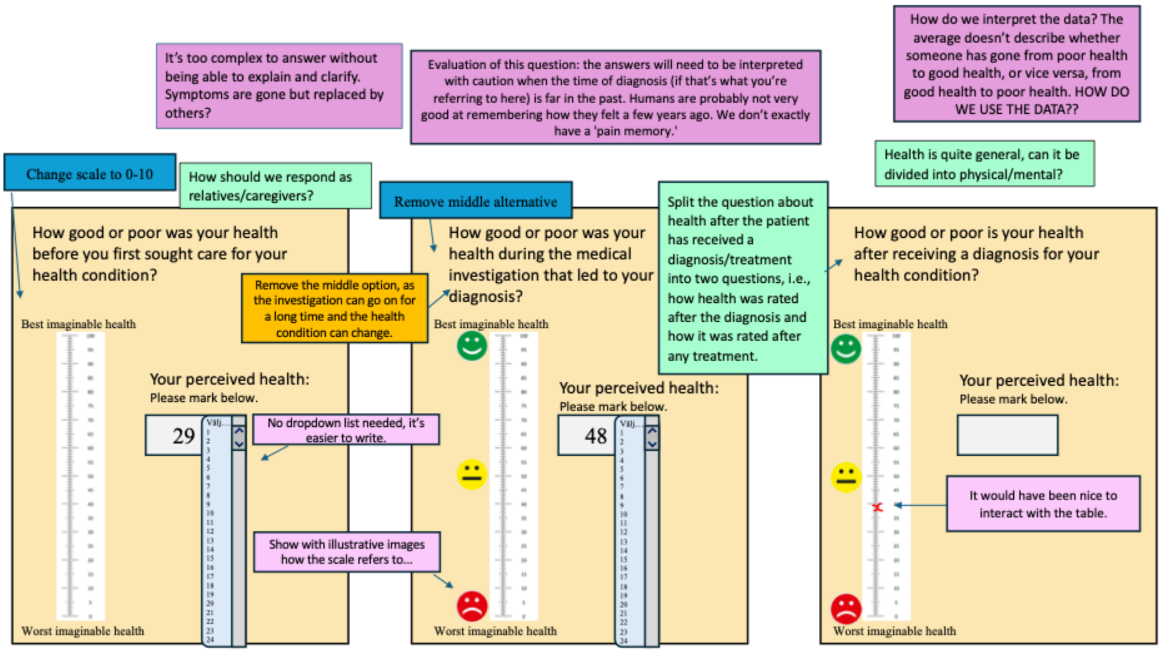

Figure S6A. Infographic During Define Phase.

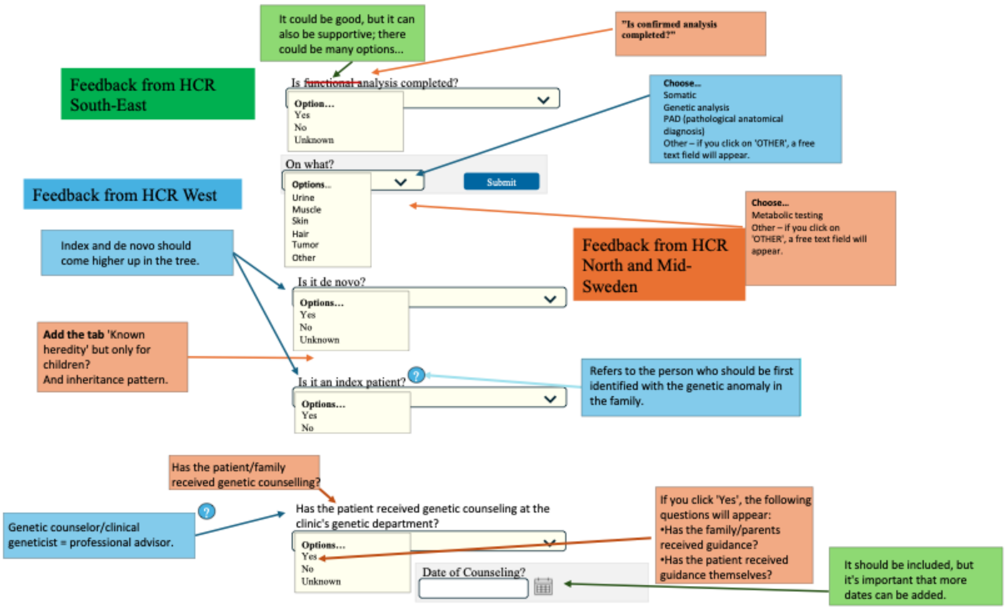

Figure S6B. Infographic of Registry Interface Prototype and Feedback Received from different Healthcare Regions (HCR)

Step 3: Ideate

The ideation phase is about generating many ideas and exploring different solution possibilities. The goal is to create a widespread flow of ideas and maximise the group's creativity without immediately judging the feasibility of the ideas.

Methods:

- Brainstorming and visualization: We produced a variety of sketches and ideas to explore various aspects of the problem and generate solutions of varying degrees.
- Creative questions and scenarios: By formulating challenges and simulating situations in the product's use cases, we generated new solution ideas.
- Iterative reflection: During the ideation phase, new insights led us to revisit and redefine earlier sketches. It is important to be prepared to revise and improve previous steps. (Figure 7.)

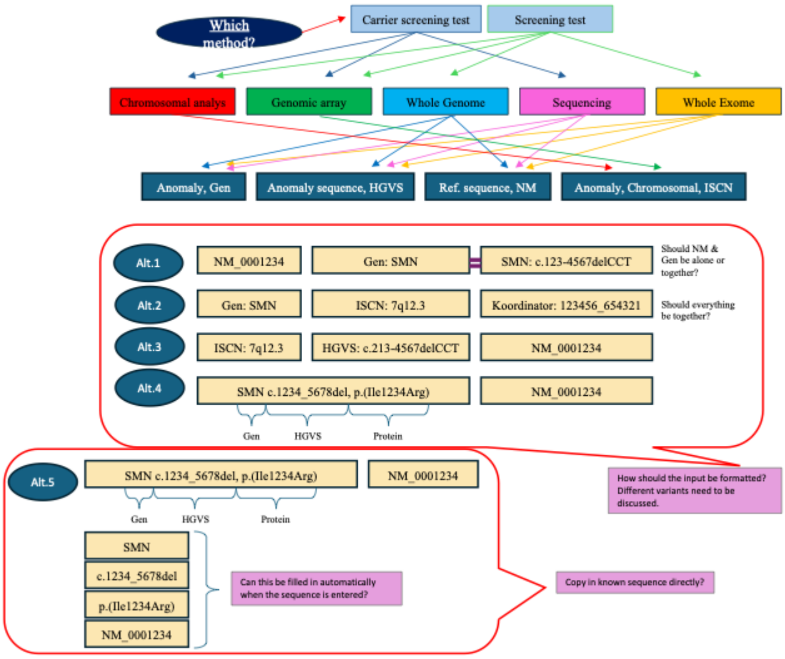

Figure S7A. Genetic Data Input and Analysis Method Ideation During RaraSwed

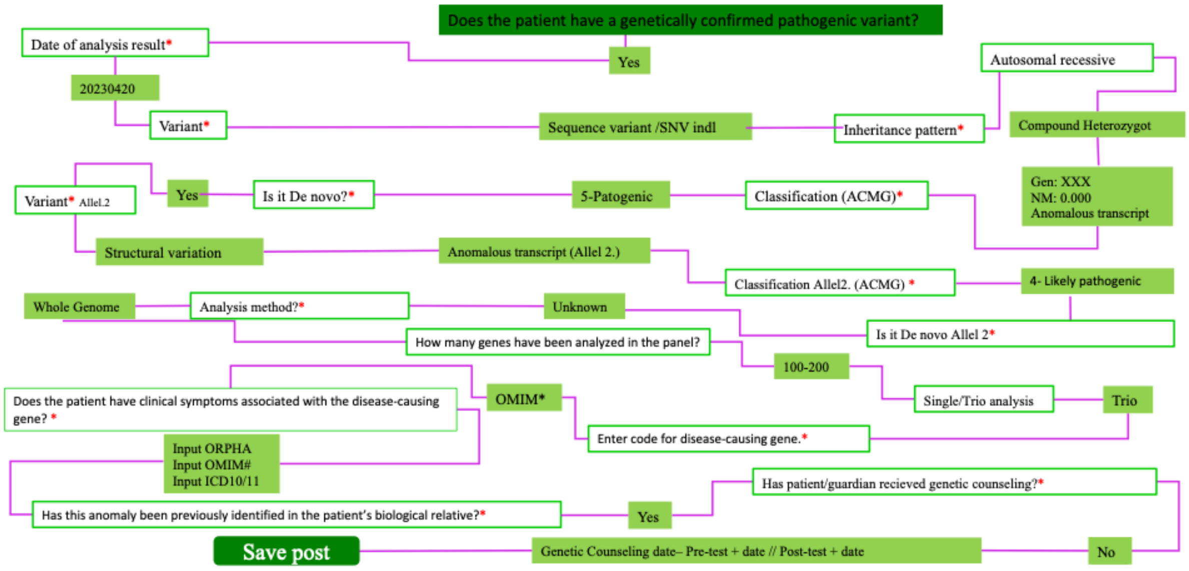

Figure S7B. Data Input Pathway Ideation during RaraSwed.

Step 4: Prototype
Prototype development involves bringing ideas to life in either physical or digital form. The goal is to quickly test and further develop design concepts by creating interactive and simple models that users can interact with.
Methods:
Empathy Prototyping: This method involves creating quick prototypes to better understand the user's feelings and experiences. This provides valuable feedback, which can lead to further design iterations.
Visualise and Discuss: The images/interactions allowed participants to describe their experiences with the prototypes, helping us identify areas for improvement.
Simulating User Experiences: We simulated user situations to better understand how the product can be improved in relation to the user’s real needs and challenges. (Figure 8.)

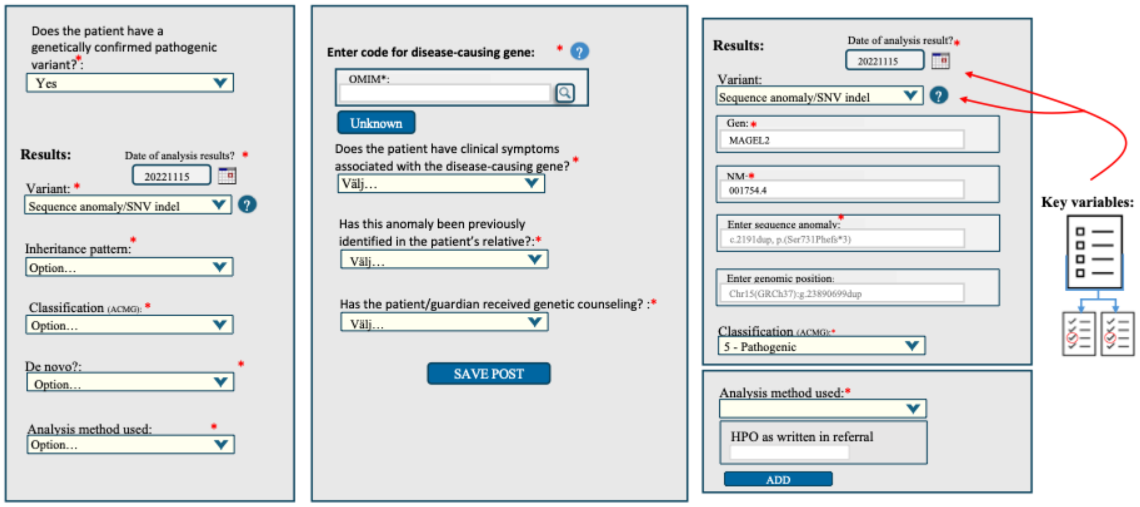

Figure S8. End-User Simulation during Prototype Phase for RaraSwed

Step 5: Test
The testing phase is critical for validating and refining the prototype based on user feedback. In our testing environment, we animated many of the features in PowerPoint to give the most realistic representation of the product. Testing also provides insights into whether the problem has been correctly defined and enables adjustments to better meet the user’s needs.

Methods:
Feedback from Users and Steering Group: The prototypes were tested both internally and with users to gather opinions for adjustments.
Modelling Design Alternatives: By testing distinct parts of the prototypes, we were able to focus on specific design parameters and improve and update the version.
Reflect and Adjust: Testing allows the problem statement to be reformulated if new insights arise, which can lead to a more user-tailored final product. This reflects the iterative design process. (Figure 9.)

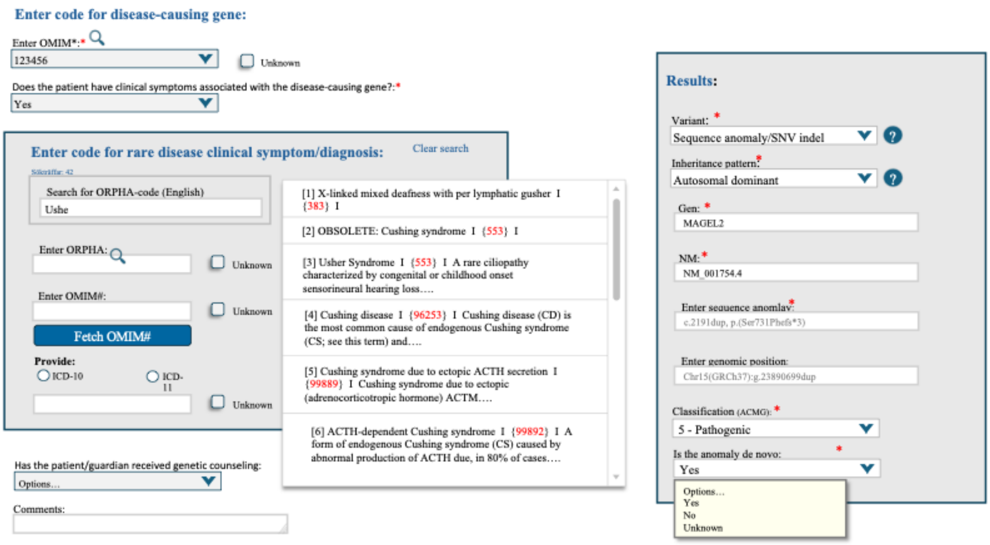

Figure S9. Testing of Prototype of RaraSwed

Summary

The iterative and user-centred Design Thinking process created a flexible structure for continuously collecting, processing, and implementing feedback. By integrating insights from users and the steering group with visual and practical methods for idea development, we were able to offer a more robust and adaptive process for developing a NQR. This process demonstrates how both the end-user and caregiver needs have been considered and accounted for in the final product. This also demonstrates how the final product at the same time is focused on overcoming challenges and maximising the product’s usability.

Integration of "React" Blocks into the Design Process:

To further enhance and adapt the five core steps of Design Thinking (DT), we added an integrated element to the process called "React" (Figure 9.). The "React" blocks are not a separate method, but a set of cycles applied between the five main steps of DT. The purpose of these blocks is to create a structured transition between phases, where insights and lessons from one phase are carried into the next, ensuring continuous improvement and refinement of the design solutions. Below is an overview of the purpose and functions of the different "React" blocks.

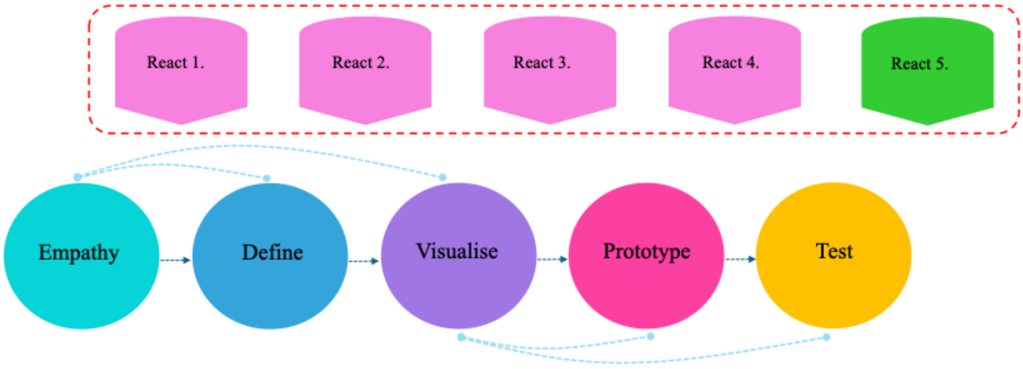

Figure S10. Modified Design Thinking Process During RaraSwed, by Dan Hellström

Description of the "React" Blocks
1. Map Patterns
The first reaction block involves mapping the patterns and insights identified during the empathy phase. By visualising and grouping the information—such as through colour coding, photography, and sketching—we can identify key needs and behaviours of the users. Mapping also enables a structured analysis of similarities and differences between various products, services, or use cases.
Purpose: Mapping patterns facilitates a clearer understanding of user needs and insights, which is crucial for a user-centred design process.
2. Communicate
After the Define phase, there will be a need to communicate the collected knowledge to the team. By presenting key insights and user needs, a foundation is created for a shared understanding and a unified direction in the design work. This step enables discussions and the identification of specific challenges, which in turn leads to the development of solutions that are relevant to the user's actual needs.
Purpose: Communicating insights and needs supports a shared cognitive framework within the team, which is a fundamental aspect of collaboration and action.
3. Concretise
After the ideation phase, where various solutions have been explored, the process moves to concretisation. This block involves transforming abstract ideas into concrete, visual concepts without immediately eliminating solutions based on technical, legal, or financial barriers. The purpose here is to maintain a broad range of potential solutions that can meet the user's needs.
Purpose: Concretising ideas after a creative phase ensures that ideas are translated into understandable and usable concepts, which promotes decision-making and user engagement.
4. Refine the Product
In the refinement block, work focuses on details/parts and the interactive aspects of the product. This block is particularly important for simulating and observing how users interact with the product in practice. By observing and analysing reactions and engagement during simulated scenarios, the team can identify new perspectives and potential improvements that might otherwise have been overlooked.
Purpose: Refinement is a central part of the iterative design process and allows for a user-friendly approach to ensure an optimal experience.
5. Finalise
Finally, the process reaches a stage where a decision can be made about whether the design meets the defined goals and can move on to the next step in the design process. Here, it is important to balance efficiency to avoid getting stuck, while maintaining flexibility for future events in the process that may affect the decision.
Purpose: By making decisions based on insights and feedback from previous cycles, the team can act effectively without compromising the design's quality.

Summary of the "React" Blocks
Integrating the "React" blocks into the DT process aims to create a continuous flow of insights, feedback, and refinement between the five main phases. These reaction blocks allow new knowledge to be smoothly carried from one phase to the next, ensuring that the decision of product design remains aligned with user needs throughout the development cycle. By following this structured, iterative process, the risk of losing important insights is reduced, and the team can work in a flexible, transparent, and user-centred way.

Design Process Implementation
The overall design process can be visualised in a schematic diagram that encompasses four main phases: Empathy, Design, Construction, and Information Structure (Figure 10). We have chosen to call each phase a “Sprint”. These sprints illustrate not only our work methodology but also the chain of tasks performed during the development of the first part of the NQR. The diagram shows how the different sprints overlap, and how we can return to a previous sprint/phase if new challenges arise. This is a natural part of DT, where continuous feedback can influence several aspects of the project simultaneously.

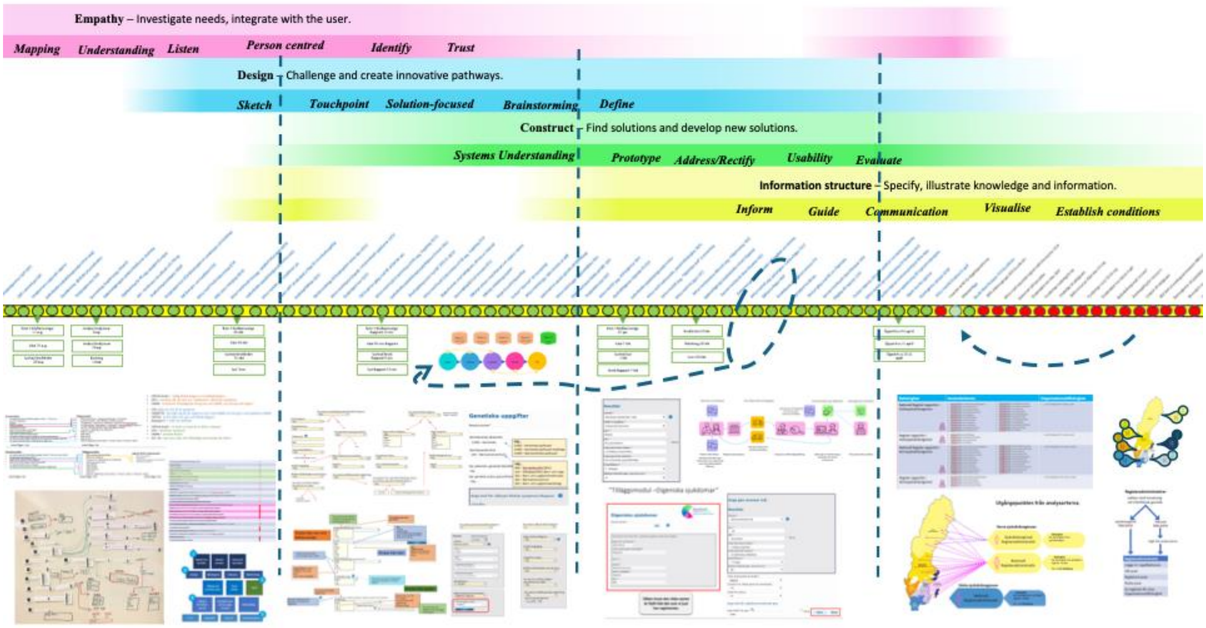

Figure S11. Schematic overview of the Design Thinking Process During RaraSwed, including the four main phases: Empathy, Design, Construction, and Information Structure.

We previously described the different methods and tools that can be used in creating the registry. The section below describes the four overlapping sprints. Sprints 1-3 have been discussed above. The fourth sprint, which we have named “Information Structure”, refers to the work involving information, guidance, visualisation, and creating the conditions for using the registry. This phase has run in parallel with much of the work and has been an important part of spreading the implementation and understanding of the registry. The challenges of designing variables, interfaces, interactive solutions, and connections are a key aspect, but the informational aspect has also required its own design solutions. In this sprint, we consciously used the same methods by illustrating scenarios and sketches to visualise the complex relationships and challenges. This contributed to a smoother and more effective implementation of the registry.

Sprints in the Design Process
1. Empathy
The empathy phase is the human-centred and exploratory phase, where the focus is on understanding users’ needs and context. A deep understanding and trust are established here, forming the foundation for constructive feedback during the process. While the empathy phase is most prominent at the beginning, it continues to play a vital role in later phases, especially in user testing and information cycles. There’s a need to listen to the users' needs again to create the conditions for a user-centred outcome.
2. Design

The design phase is characterised by a higher degree of creativity, where various perspectives and solution strategies are explored. Through mapping, brainstorming, and sketching, key touchpoints and solution models are identified based on insights from the empathy phase. This promotes a deeper understanding of the requirements and opportunities that can enhance the user experience.

3. Construction

In the construction phase, ideas are transformed into concrete solutions by systematically integrating technical knowledge and experience from prototype testing. This involves a detailed examination of features and usability, where the product is assessed from a holistic perspective. Through this process, potential issues can be identified and addressed, ensuring that the design is functional and usable for the end user.

4. Information Structure

The role of information structure emerges in the later part of the design process, where the focus is on creating a cohesive communication system and guidance for the product. Here, logistics and infrastructure around the product are integrated to ensure accessibility and usability. This phase requires responsiveness to user needs and aims to clarify and facilitate the product for future use. The cycle also appears earlier in the process regarding clarifying needs, expectations, and structure for further development of the product.

By using a structured and iterative design process that integrates these phases, the project can adapt to changing requirements and create a more sustainable outcome. This holistic approach has facilitated a smooth and effective implementation of the registry while improving its functionality and user experience.

Author: Dan Hellström, 25th of January 2025
