# Supplementary material for: Extracting homogenous data from heterogenous diseases: RaraSwed, the Swedish national rare disease quality registry
Source: BMC Glob Public Health. 2026 Jun 16;4:58. doi: 10.1186/s44263-026-00276-9 (PMC13270570; doi:10.1186/s44263-026-00276-9)
Supplement: Supplementary file 3 — Supplementary Materials 3 Title: Glossary of Terms. Description: This glossary serves as a reference for the core terminology used throughout this work and is divided into two distinct sections. The first section contains a translation guide providing English-to-Swedish translations for key health organisation and administrative terms, while the second section offers a comprehensive health system strengthening glossary adapted from the World Health Organization Glossary List to provide necessary context for the systemic aspects of the RaraSwed implementation [file 44263_2026_276_MOESM3_ESM.pdf]

Supplementary Materials 3: Glossary of Terms

Translation of Health Organisation Terms from English to Swedish
Centre of Rare Disease = Centrum för Sällsynta Diagnoser
Central Data Protection Authority = Centralt personuppgiftsansvarig myndighet, CPUA-myndigheten
Collaboration = Samverkan
Consensus = Samsyn
National Quality Policy and Strategy = Folkhälsopolitik
Health Equity = Jämlik vård
Healthcare Regions in Collaboration Group = Sjukvårdsregioner i Samverkan
National System for Knowledge-driven Management (NSKM) = Kunskapsstyrning
County council/region = Region
National program area = Nationella programområde
Knowledge-Driven Management for Rare Disease (KDM RD) = Kunskapstyrningen för sällsynta diagnoser, Nationella programråde (NPO) sällsynta diagnoser
Knowledge-Driven Management for Rare Disease (KDM RD) National Committee = Ordförande och ledamöter för Nationella programområde sällsynta diagnoser.
Regional rare disease working group = Regionalt programområde sällsynta diagnoser
Local rare disease working group = Lokalt/kommunalt programområde sällsynta diagnoser
Healthcare Region Federation
Registry Steering Committee = Registerstyrgrupp
Registry Holder = Registerhållare
Expert Group = Sakkunniggrupp
Patient advocacy (representative) group = Patientförening
Regional Cancer Centre = Regionalt cancercentrum
Registry Centre Organisation = Registercentrum
National Quality Registry (NQR) of Rare Disease = Nationella kvalitetsregister (NKR) för sällsynta diagnoser
Healthcare region (HCR) = Sjukvårdsregion
The National Board of Health and Welfare = Socialstyrelsen
Standardisation of cancer care pathways = Standardiserade vårdförlopp cancer
Strategic operational plan = Strategisk utvecklingsplan
Swedish Association of Local Authorities and Regions (SALAR) = Sveriges Kommuner och Regioner
National clinical care guidelines = Vårdprogram

Source: Adapted from Regional Cancer Centrum Glossary, modified to include rare disease national and regional entities. Accessed 8 Nov 2024: https://cancercentrum.se/stockholm-gotland/vara-uppdrag/ledning-och-styrning/comprehensive-cancer-centre/tips-och-rad/ord-och-begrepp-pa-engelska/

Glossary List Adapted from World Health Organization, Health Systems Strengthening Glossary

Burden of disease: a measurement of the gap between current health status and an ideal situation where everyone lives into old age, free of disease and disability.[1]

Clinical Practice Guidelines: systematic recommendations, based on the best available scientific knowledge, to guide the decisions of both professionals and patients regarding the most appropriate, efficient health interventions for addressing a specific health-related problem given specific circumstance.[1]

Decentralisation: political reform designed to promote local autonomy, decentralization entails changes in authority and financial responsibility for health services. Hence, decentralization can have a large impact on health service performance. There are several forms of decentralization affecting the health sector in different ways: (i) deconcentration, which transfers authority and responsibility from the central level of the Ministry of Health to its field offices; (ii) delegation, which transfers authority and responsibility from the central level of the Ministry of Health to organizations not directly under its control; (iii) devolution, which transfers authority and responsibility from the central level of the Ministry of Health to lower level autonomous units of government; (iv) privatization, which involves the transfer of ownership and government functions from public to private bodies, which may consist of voluntary organizations and for-profit and not-for-profit private organizations, with varying degree of government regulation.[1]

Equity in Health: (i) the absence of systematic or potentially remediable differences in health status, access to healthcare and health-enhancing environments, and treatment in one or more aspects of health across populations or population groups defined socially, economically, demographically, or geographically within and across countries; (ii) a measure of the degree to which health policies are able to distribute well-being fairly.[1]

Essential Public Health Functions: the health authority's functions with regard to: (i) monitoring, evaluation and analysis of health status; (ii) surveillance, research and control of the risks and threats to public health; (iii) health promotion; (iv) social participation in health; (v) development of policies and institutional capacity for public health planning and management; (vi) strengthening of public health regulation and enforcement capacity; (vii) evaluation and promotion of equitable access to necessary health services; (viii) human resources development and training in public health; (ix) quality assurance in personal and population-based health services; (x) research in public health; and (xi) Reduction of the impact of emergencies and disasters on health.[1]

Evidence: "any form of knowledge, including, but not confined to research, of sufficient quality to inform decisions".[1]

Fragmentation (of health services): (i) coexistence of units, facilities, or programmes that are not integrated into the health network; (ii) services that do not cover the entire range of promotion, prevention, diagnosis, treatment, rehabilitation, and palliative care services; (iii) services at different levels of care that are not coordinated among themselves; (iv) services that do not continue over time; (v) services that do not meet people's needs.[1]

Governance: (i) the exercise of political, economic, and administrative authority in the management of a country's affairs at all levels, comprising the complex mechanisms, processes, relationships and institutions through which citizens and groups articulate their interests, exercise their rights and obligations and mediate their differences, (ii) the traditions and institutions by which authority in a country is exercised for the common good, including the processes by which those in authority are selected, monitored and replaced; the capacity of the government to effectively manage its resources and implement sound policies; and the respect of citizens and the state for the institutions that govern economic and social interactions among them, (iii) the process of creating an organizational vision and mission – what it will be and what it will do - in addition to defining the goals and objectives that should be met to achieve the vision and mission; of articulating the organization, its owners and the policies that derive from these values - policies concerning the options that its members should have in order to achieve the desired outcomes; and adopting the management necessary for achieving those results and a performance evaluation of the managers and the organization as a whole.[1]

Harmonisation: the coordination of donors' contributions and activities, the transparent sharing of information and the attempt to be collectively effective and avoid duplication. See Paris Declaration.[1]

Health planning: (i) the orderly process of defining health problems, identifying unmet needs and surveying the resources to meet them, establishing priority goals that are realistic and feasible, and projecting administrative action, concerned not only with the adequacy, efficacy and efficiency of health services but also with those factors of ecology and of social and individual behaviour that affect the health of the individual and the community", (ii) the process of organizing decisions and actions to achieve particular ends, set within a policy, (iii) a code word for public decision making towards the future, often used interchangeably with policy formation or developing strategies and programmes.[1]

Health policy: (i) a set of decisions or commitments to pursue courses of action aimed at achieving defined goals for improving health, stating or inferring the values that underpin these decisions; the health policy may or may not specify the source of funding that can be applied to the action, the planning and management arrangements to be adopted for implementation of the policy, and the relevant institutions to be involved, (ii) a general statement of understanding [to] guide decision making that results from an agreement or consensus among relevant partners on the issues to be addressed and on the approaches or strategies to deal with them.[1]

Health Sector Reform: (i) "a movement aimed at reconfiguring health services, dominant in the 90s in the framework of the New Public Management, typically including the following components: separating the roles of financing and provision and the possible introduction of a managed market; developing alternative financing

mechanisms, particularly user charges and health insurance; decentralization; limiting the public sector and encouraging a greater role for the private sector; prioritizing the use of cost-effectiveness techniques”.[1]

Health service: any service (i.e. not limited to medical or clinical services) aimed at contributing to improved health or to the diagnosis, treatment, and rehabilitation of sick people.[1]

Health system: (i) all the activities whose primary purpose is to promote, restore and/or maintain health; (ii) the people, institutions, and resources, arranged together in accordance with established policies, to improve the health of the population they serve; while responding to people’s legitimate expectations and protecting them against the cost of ill-health through a variety of activities whose primary intent is to improve health.[1]

Health system building blocks: an analytical framework used by WHO to describe health systems, disaggregating them into 6 core components: leadership and governance (stewardship), service delivery, health workforce, health information system, medical products, vaccines and technologies, and health system financing.[1]

Health systems strengthening: (i) The process of identifying and implementing the changes in policy and practice in a country’s health system, so that the country can respond better to its health and health system challenges; (ii) any array of initiatives and strategies that improves one or more of the functions of the health system and that leads to better health through improvements in access, coverage, quality, or efficiency.[1]

National disease/programme strategy or national disease/programme strategic plan: strategic plan to guide the control of a particular disease or health problem at national level, with the intended actions to achieve the goals of a given programme. Ideally aligned to the national health strategic plan.[1]

National health strategy, also known as a national health strategic plan or national health plan: a process of organizing decisions and actions to achieve particular ends, set within a policy, providing “a model of an intended future situation and a programme of action predetermined to achieve the intended situation”. Refers to the broad, long term lines of action to achieve the policy vision and goals for the health sector, incorporating “the identification of suitable points for intervention, the ways of ensuring the involvement of other sectors, the range of political, social, economic and technical factors, as well as constraints and ways of dealing with them”.[1]

Operational plan: operational plans focus on effective management of resources with a short time framework, converting objectives into targets and activities, and arrangements for monitoring implementation and resource usage. Specific meanings include: (i) translation of the national strategic plan within a one-year time frame; (ii) translation of the national strategic plan into a sub-national plan, e.g. a district plan, usually with a shorter time frame than the national strategic plan; (iii) a subset of a national strategic plan, limited to a particular programme.[1]

Policy dialogue: (i) the process of policy making or policy formation, i.e. of recognition of social demand, transformation into political demand and, eventually, into formulation of a policy statement that provides guidance to subsequent decisions about technical implementation and/or (ii) the social debate and interaction between stakeholders that leads to translation of policy into strategies and plans.[1]

Primary care: often used interchangeably with first level of care. (i) the part of a health services system that assures person focused care over time to a defined population, accessibility to facilitate receipt of care when it is first needed, comprehensiveness of care in the sense that only rare or unusual manifestations of ill health are referred elsewhere, and coordination of care such that all facets of care (wherever received) are integrated. Quality features of primary care include effectiveness, safety, people-centredness, comprehensiveness, continuity, and integration; (ii) the provision of integrated, accessible health care services by clinicians who are accountable for addressing a large majority of personal health care needs, developing a sustained partnership with patients, and practicing in the context of family and community.[1]

Public Health: an organized effort by society, primarily through its public institutions, to improve, promote, protect, and restore the health of the population through collective action. It includes services such as health situation analysis, health surveillance, health promotion, prevention, infectious disease control, environmental protection and sanitation, disaster and health emergency preparedness and response, and occupational health, among others.[1]

Quality assurance: All the planned and systematic activities implemented within the quality system, and demonstrated as needed, to provide adequate confidence that an entity will fulfil requirements for quality. Quality improvement: “An organizational strategy that formally involves the analysis of process and outcomes data and the application of systematic efforts to improve performance”.[2]

Quality management: All activities of the overall management function that determine the quality policy, objectives, and responsibilities, and implement them by means such as quality planning, quality control, and quality improvement within the quality system.[3,4]

Situation analysis: analysis of the current status and expected trends in a country's health and health system. Ideally includes: (i) assessment of current and future health needs and determinants of health; (ii) assessment of expectations and demand of services; (iii) assessment of the health system performance, health sector capacity and health system resources, and the gaps in responding to current and future needs and expectations; and analysis of stakeholder positions.[1]

Stakeholder: an individual, group or an organization that has an interest in the organization and delivery of health care.[1]

Strategy: a series of broad lines of action intended to achieve a set of goals and targets set out within a policy or programme.[1]

Systems thinking: is an approach to problem solving that views "problems" as part of a wider, dynamic system. Systems thinking involves much more than a reaction to present outcomes or events. It demands a deeper understanding of the linkages, relationships, interactions, and behaviours among the elements that characterize the entire system. Commonly used in other sectors where interventions and systems are complex, systems thinking in the health sector shifts the focus to:

- the nature of relationships among the building blocks
- the spaces between the blocks (and under- standing what happens there)
- the synergies emerging from interactions among the blocks.

The application of systems thinking in the health sector is accelerating a more realistic under- standing of what works, for whom, and under what circumstances.[5]

Universal coverage: universal access to health services with social health protection.[1]

References:

1 World Health Organization. Geneva. Health systems strengthening glossary. www.who.int/docs/default-source/documents/health-systems-strengthening-glossary.pdf (accessed 12 Nov 2024).

2 Gliklich RE, Leavy MB, Dreyer NA. Registries for Evaluating Patient Outcomes: A User’s Guide. 4th ed. 2014 doi:10.23970/AHRQEPCREGISTRIES4.

3 Bannock G, Brexter RE, Davis E. *Dictionary of economics*. The Economist Books: London, 1998.

4 Hoeijmakers F, Beck N, Wouters MWJM, Prins HA, Steup WH. National quality registries: how to improve the quality of data? *J Thorac Dis* 2018; **10**: S3490–S3499.

5 Savigny D de, Adam T, World Health Organization. Systems thinking for health systems strengthening. 2009.
